# Supplementary material for: Palmitic acid in type 2 diabetes mellitus promotes atherosclerotic plaque vulnerability via macrophage Dll4 signaling
Source: Nat Commun. 2024 Feb 12;15:1281. doi: 10.1038/s41467-024-45582-8 (PMC10861578; doi:10.1038/s41467-024-45582-8)
Supplement: Supplementary file 1 — Supplementary Information [file 41467_2024_45582_MOESM1_ESM.pdf]

Supplementary Information for

Palmitic Acid in Type 2 Diabetes Mellitus Promotes Atherosclerotic Plaque Vulnerability via Macrophage Dll4 Signaling

Xiqiang Wang[#1], Ling Zhu[#1], Jing Liu[1], Yanpeng Ma[1], Chuan Qiu[2], Chengfeng Liu[1], Yangchao Gong[1], Ya Yuwen[1,3], Gongchang Guan[1], Yong Zhang*[1], Shuo Pan*[1], Junkui Wang*[1], Zhongwei Liu*[1,4]

[1] Department of Cardiology, Shaanxi Provincial People's Hospital, Xi'an, Shaanxi Province, 710068, China

[2] Division of Bioinformatics and Genomics, Deming Department of Medicine, Tulan Center of Biomedical Informatics and Genomics, Tulane University, New Orleans, LA, 70112, USA

[3] Medical School, Xizang Minzu University, Xianyang, Shaanxi Province, 712000, China

[4] Affiliated Shaanxi Provincial People's Hospital, Medical Research Institute, Northwestern Polytechnical University, Xi'an, Shaanxi Province, 710072, China

* Correspondence should be addressed to Z.L. (medicalman@163.com), S.P. (panshuosx@163.com), J.W. (junkuiwang@yeah.net) and Y.Z. (zhangyong971292@163.com)

Table of contents

Supplementary figures……………………………………………………..…3-28

Figure S1………………………………………………………………………...3

Figure S2………………………………………………………………………...4

Figure S3………………………………………………………………………...5

Figure S4………………………………………………………………………...6

Figure S5………………………………………………………………………...7

Figure S6………………………………………………………………………...8

Figure S7……………………………………………………………………...9
Figure S8…………………………………………………………………......10
Figure S9………………………………………………………...……………...11
Figure S10………………………………………………...…………………12
Figure S11………………………………………………...…………………13
Figure S12…………………………………………...…………………....14
Figure S13…………………………………………...……………………15
Figure S14…………………………………………...…………………16-17
Figure S15…………………………………………...……………………18
Figure S16…………………………………………...……………………19
Figure S17…………………………………………...……………………20
Figure S18…………………………………………...……………………21
Figure S19…………………………………………...……………………22
Figure S20…………………………………………...……………………23
Figure S21…………………………………………...…………………24-25
Figure S22…………………………………………...……………………26
Figure S23…………………………………………...……………………27
Figure S24…………………………………………...……………………28
Supplementary tables…………………………………...……………………29-44
Table S1…………………………………………...……………………29-30
Table S2…………………………………………...……………………31-37
Table S3…………………………………………...……………………....38
Table S4…………………………………………...…………………....39-42
Table S5…………………………………………...…………………….....43

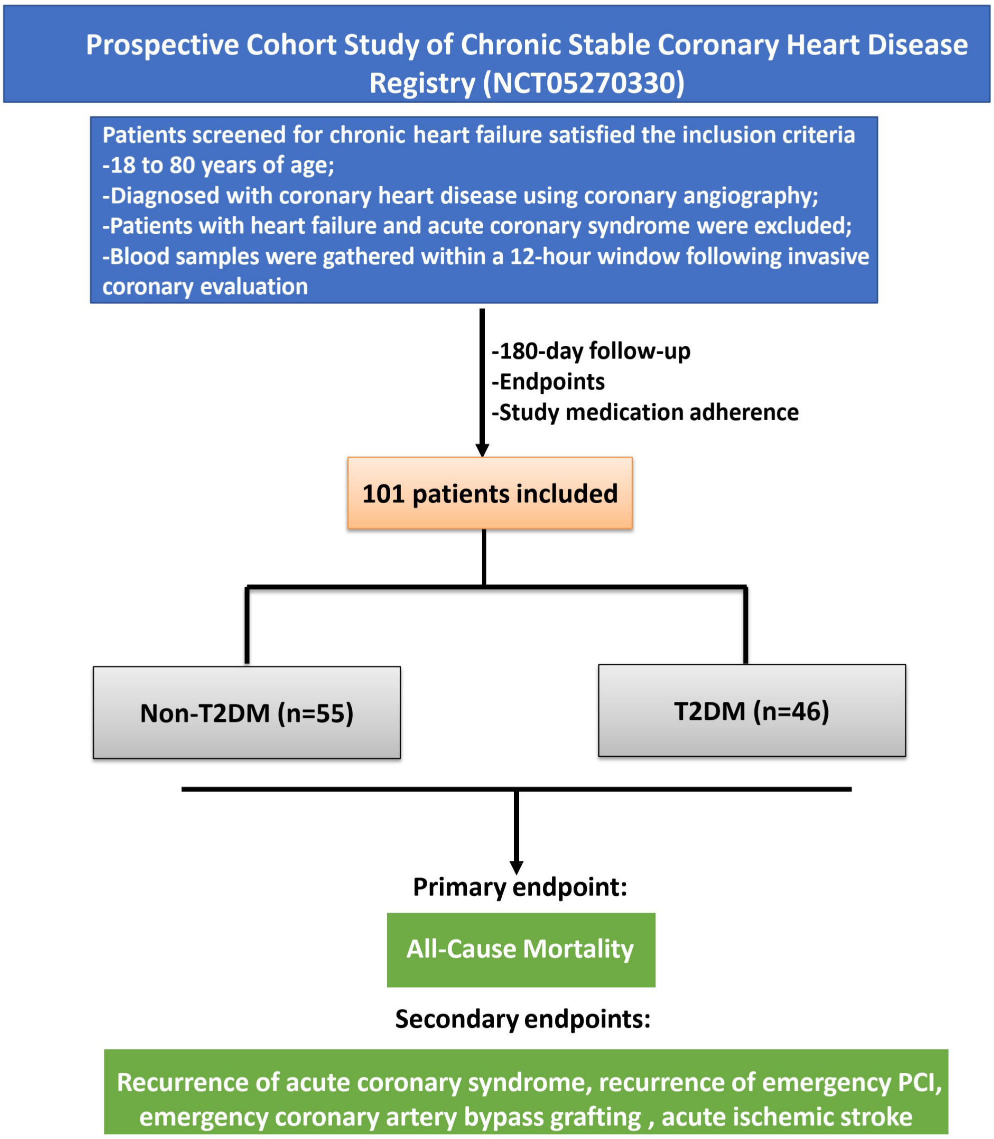

Figure S1: Flowchart of cohort study of "Chronic Stable Coronary Heart Disease" Prospective Cohort registry (Clinicaltrail.gov identifier: NCT05270330)

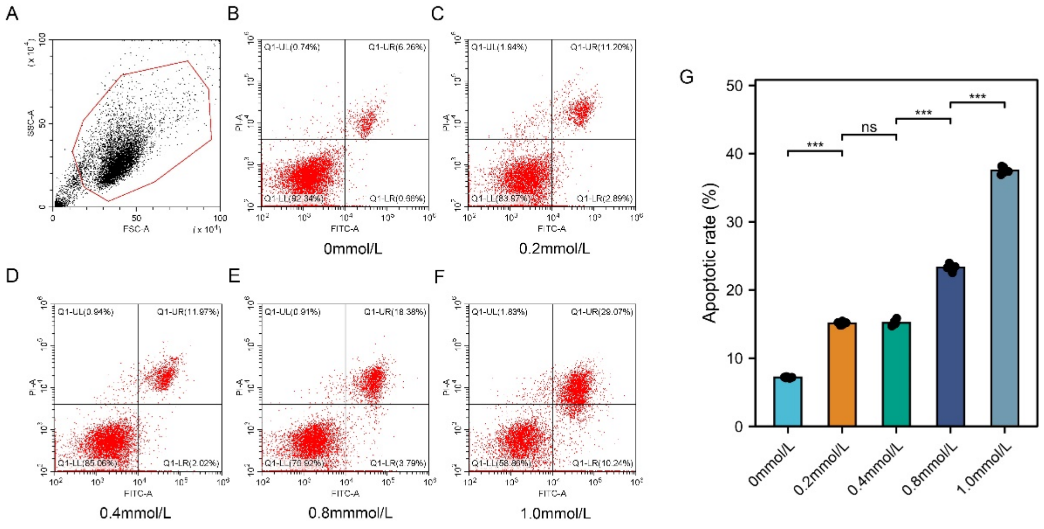

Figure S2: Determining optimal palmitic acid concentration for primary macrophage treatment

A. FSC/SSC plot showing the population of macrophages. B-F. Flow cytometry analysis illustrating the apoptosis levels in macrophages subjected to varying concentrations of palmitic acid (0, 0.2, 0.4, 0.8, and 1.0 mmol/L) for a duration of 6 hours. G. Quantitative representation of apoptosis rates as determined by flow cytometry in macrophages treated with escalating concentrations of palmitic acid (0, 0.2, 0.4, 0.8, and 1.0 mmol/L) over a 6-hour period. Non-significant differences are denoted as (ns P>0.05); significant differences are denoted as (*** P<0.001). Each condition was repeated in six independent experiments (n=6 independent replicates).

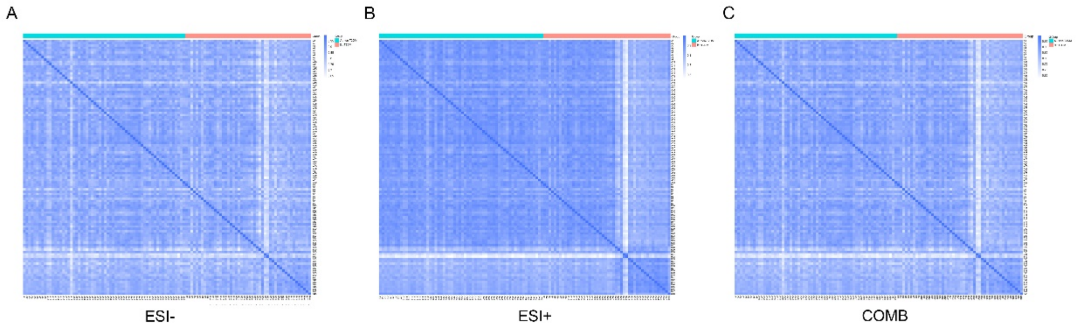

Figure S3: Quality control checks.

Pearson correlation of human serum ESI- (A), ESI+ (B) and COMB (C) QC samples. Abbreviations: ESI-, negative electrospray ionization model; ESI+, positive electrospray ionization model; COMB, combined model.

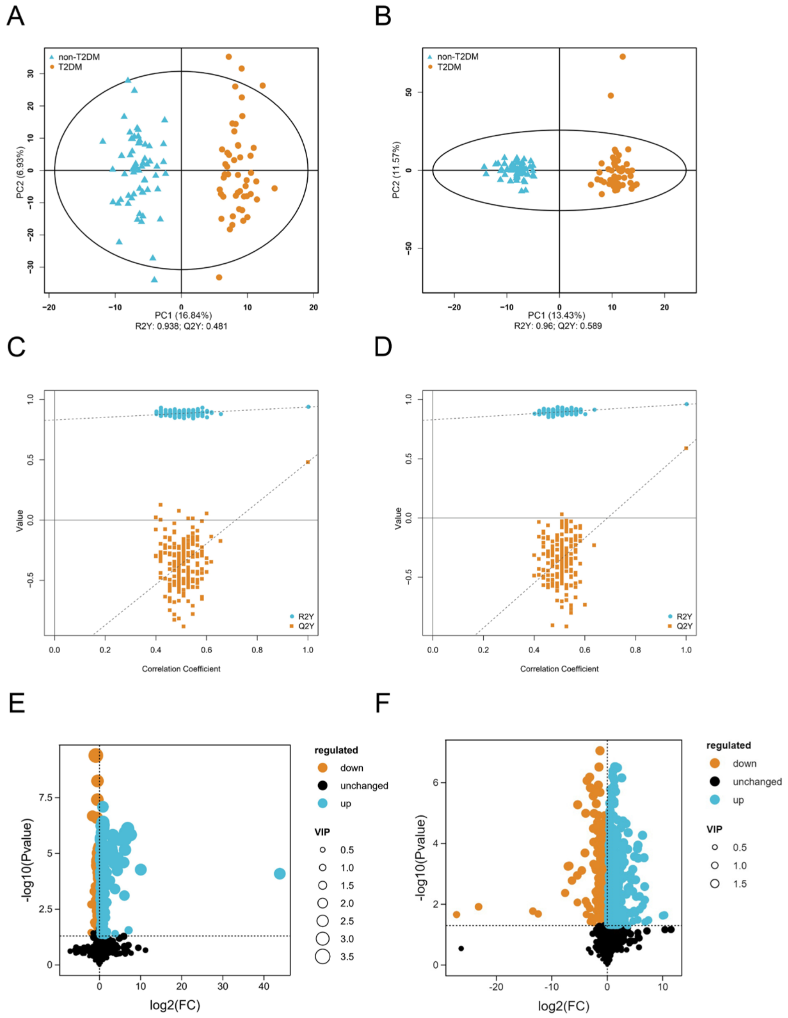

Figure S4: Identification of Metabolic Alterations in Human Cohort Serum Samples.

OPLS-DA score plots of serum samples analyzed in ESI- (A) and ESI+ (B), respectively. Validation of the OPLS-DA models via 200-times permutation tests in both ESI-(C) and ESI+ (D). The intercepts of R2 and Q2 indicate the robustness of the models and suggest the absence of overfitting. Pairwise comparison results of metabolite concentrations in T2DM versus non-T2DM samples, analyzed in ESI- (E) and ESI+ (F), indicated in volcano plots. Abbreviations: OPLS-DA, orthogonal partial least squares discriminant analysis; ESI-, negative electrospray ionization mode; ESI+, positive electrospray ionization model.

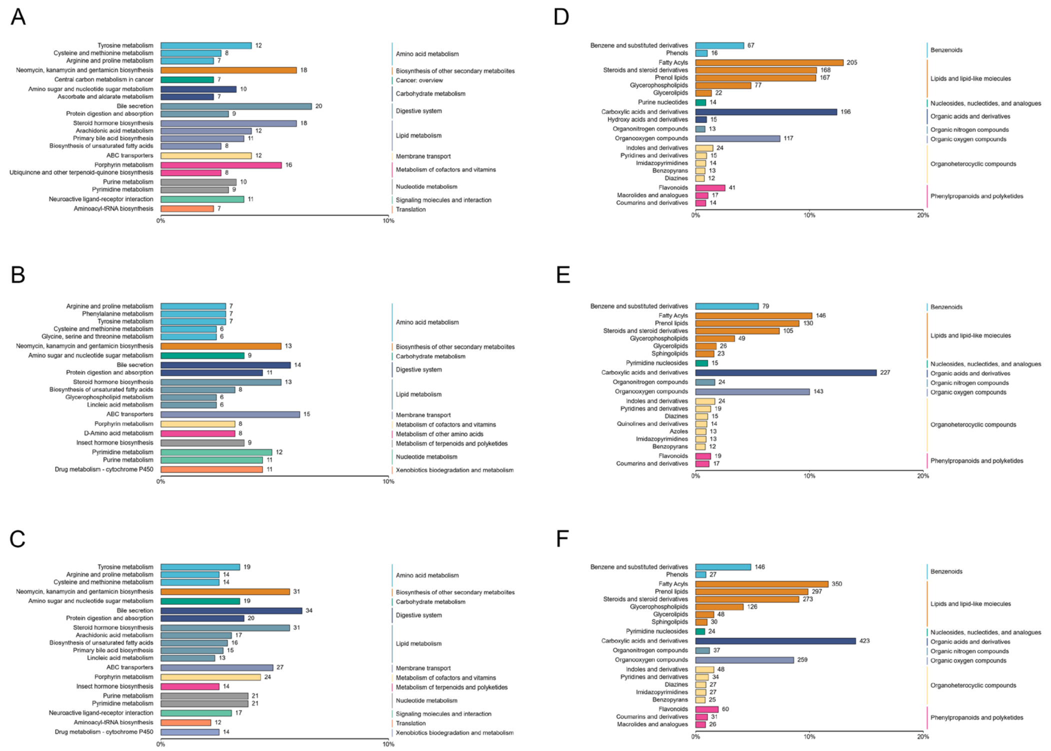

Figure S5: Differential Metabolomic Profiles in T2DM vs Non-T2DM Subjects within the Cohort

Metabolomic profiling, as identified by KEGG annotation, in T2DM versus non-T2DM subjects within the cohort for ESI- (A), ESI+ (B), and COMB (C). Metabolomic profiling, as characterized by HMDB annotation, in T2DM versus non-T2DM subjects within the cohort for ESI- (D), ESI+ (E), and COMB (F). These results illustrate distinct metabolomic patterns between T2DM and non-T2DM subjects, highlighting potential metabolic pathways involved in disease progression. Abbreviations: T2DM, type 2 diabetes mellitus; KEGG, Kyoto encyclopedia of genes and genomes; HMDB, human metabolome database; COMB, combined model.

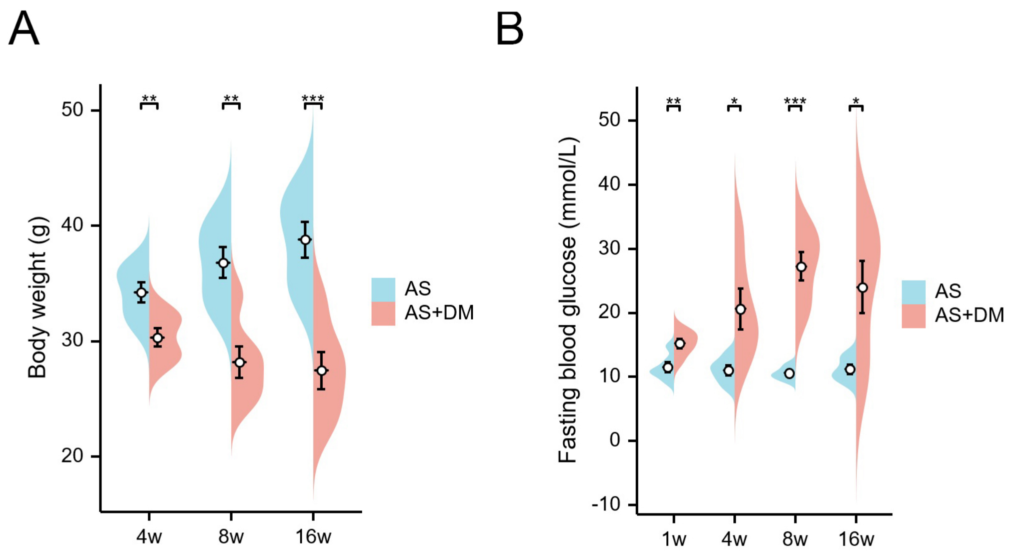

Figure S6: Tracking bodyweight and FBG alterations in atherosclerotic mice model complicated with diabetes

A. Graphic representation of mouse body weight assessments conducted at 4-week, 8-week, and 16-week intervals post STZ or vehicle control injections. B. Chart of FBG measurements recorded at 1-week, 4-week, 6-week, and 16-week periods following STZ or vehicle control injections. Statistical significance is denoted as follows: * indicates $P<0.05$; ** indicates $P<0.01$; *** indicates $P<0.001$, with each experimental group comprising 6 mice.

Abbreviations: FBG, fasting blood glucose; STZ, streptozotocin

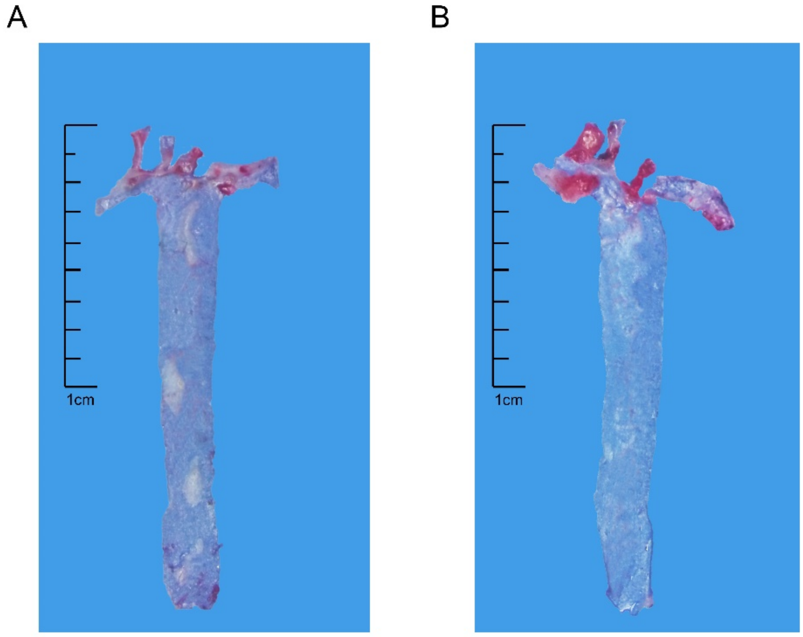

Figure S7: Visualization of Aortic Oil Red O Staining in Animal Models as a Supplement to Figure 3C

A. Representative Oil Red O staining of aorta from atherosclerosis (AS) animal models. B. representative image of aorta stained with oil red O in atherosclerosis complicated with diabetes (AS+DM) animals. Scale bar donates 1cm.

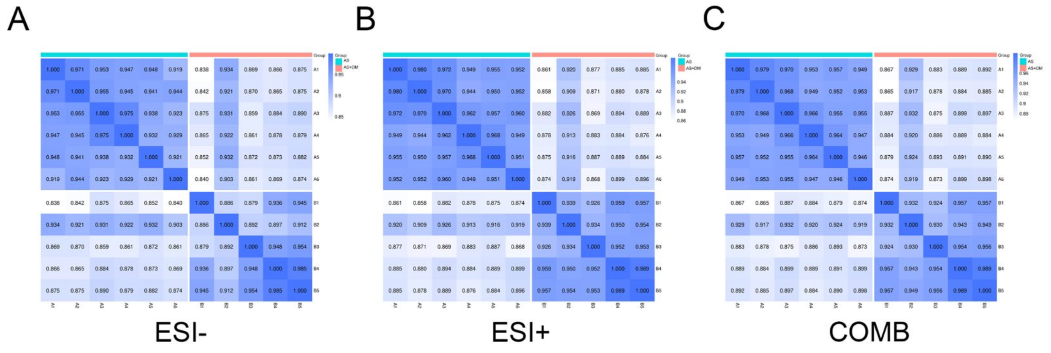

Figure S8: Quality control checks.

Pearson correlation of mice serum ESI- (A), ESI+ (B) and COMB (C) QC samples. Abbreviations: ESI-, negative electrospray ionization model; ESI+, positive electrospray ionization model; COMB, combined model.

Abbreviations: ESI-, negative electrospray ionization mode; ESI+, positive electrospray ionization model; COMB, combined model.

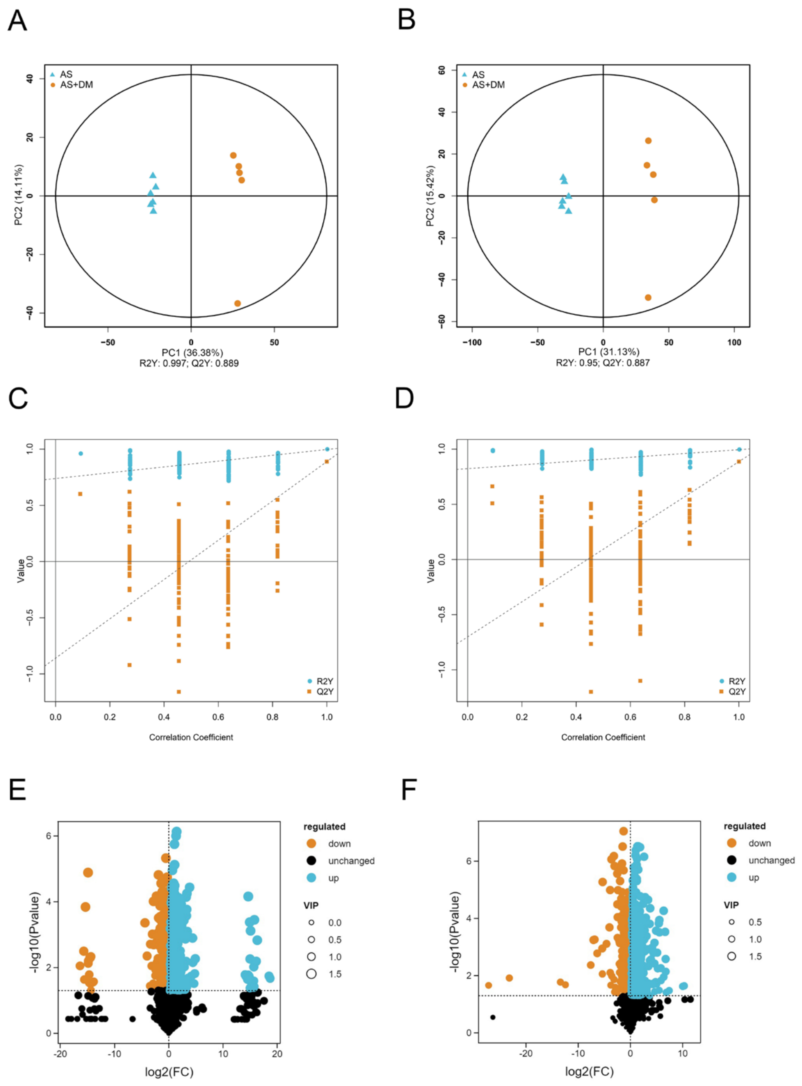

Figure S9: Elucidation of Metabolic Variations in Serum Samples of Mice. OPLS-DA score plots of murine serum samples, examined under ESI- (A) and ESI+ (B), respectively. Validation of the robustness and reliability of the OPLS-DA models using permutation tests, performed 200 times for both ESI- (C) and ESI+ (D). The intersecting coordinates of R2 and Q2 ensure the model's strength and negate the risk of overfitting. Volcano plots depicting the pairwise comparison outcomes of metabolite levels in T2DM and non-T2DM samples, assessed under ESI- (E) and ESI+ (F). Abbreviations: OPLS-DA, orthogonal partial least squares discriminant analysis; ESI-, negative electrospray ionization mode; ESI+, positive electrospray ionization model.

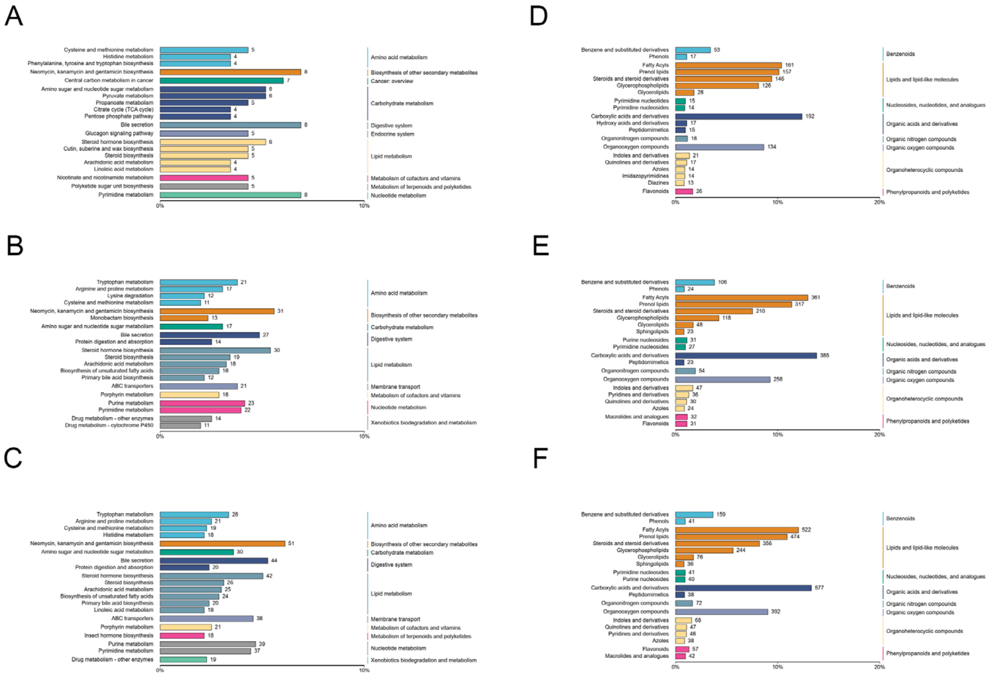

Figure S10: Comparative Metabolomic Analysis in Atherosclerosis with Diabetes (AS+DM) vs. Atherosclerosis-Only (AS) Animals.

Metabolomic profiling using KEGG annotation for ESI- (A), ESI+ (B), and COMB (C) between AS+DM and AS animals. Metabolomic characterization leveraging the HMDB annotation in ESI- (D), ESI+ (E), and COMB (F) between AS+DM and AS animals. This comprehensive metabolomic examination underlines the distinct biochemical trajectories observed in atherosclerosis complicated with diabetes (AS+DM) compared to atherosclerosis alone (AS).

Abbreviations: AS, Atherosclerosis; DM, Diabetes; KEGG, Kyoto Encyclopedia of Genes and Genomes; HMDB, Human Metabolome Database; COMB, Combined Mode.

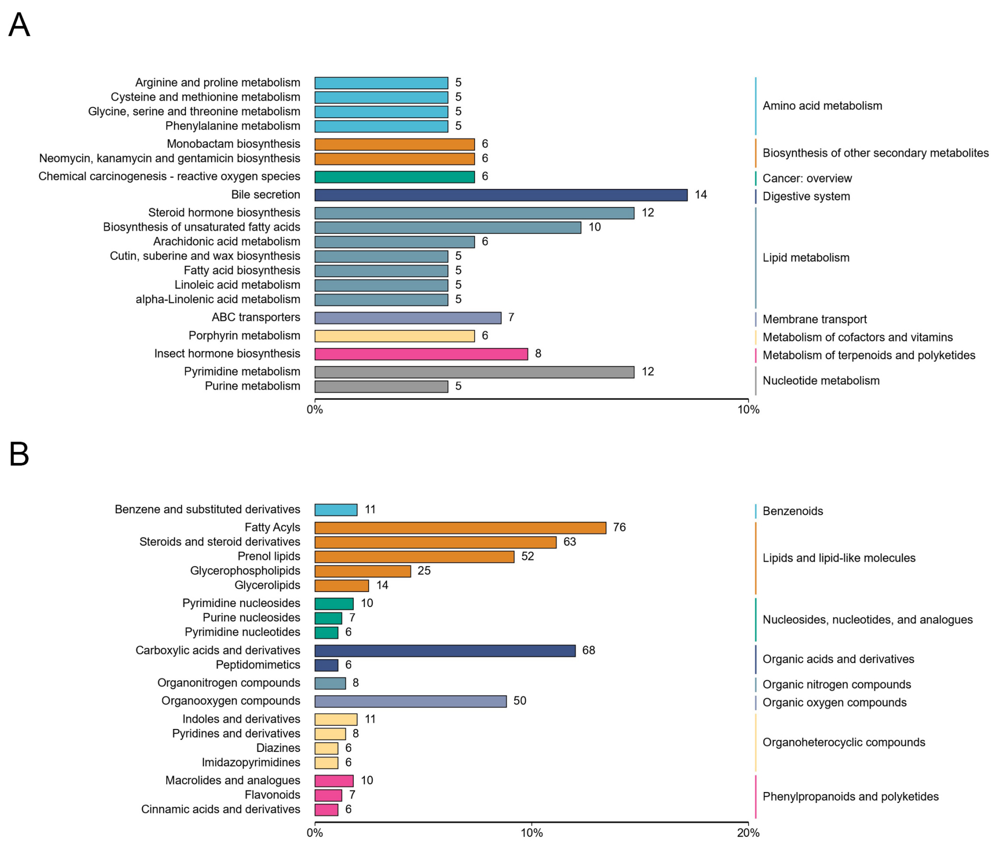

Figure S11: Integrated Metabolomic Analysis in a Unified Human-Mouse Model. In an innovative approach, we established a cohesive model that merges annotated metabolites from both human and murine cohorts, providing a comprehensive metabolomic view of diabetes-associated atherosclerosis. Specifically, "non-T2DM" and "T2DM" classifications in human subjects corresponded to "AS" and "AS+DM" in mouse models. The integrated metabolomic landscape is depicted through KEGG annotation (A) and HMDB (B) within this unified human-mouse model. Abbreviations: AS, Atherosclerosis; DM, Diabetes; KEGG, Kyoto Encyclopedia of Genes and Genomes; HMDB, Human Metabolome Database; T2DM, Type 2 Diabetes Mellitus

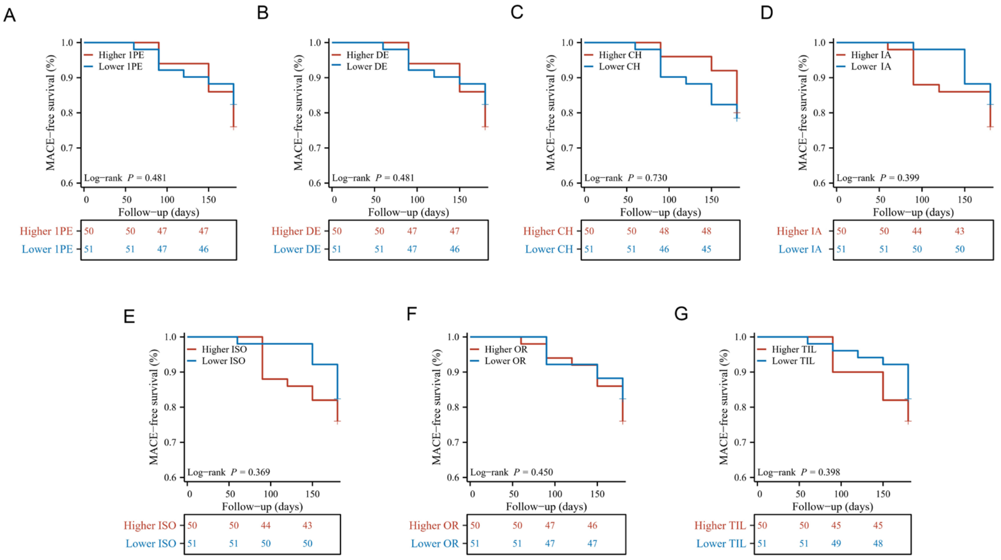

Figure S12: Analysis of association between identified metabolites and occurrences of major adverse cardiac event (MACE) in a 180-day follow-up study

Kaplan-Meier survival curve plots the 180-day MACE-free survival in the higher 1PE (A), DE (B), CH (C), IA (D), ISO (E), OR (F) and TIL (G) versus their lower counterparts in a follow-up study.

Abbreviations: 1PE, 1-pentadecanoyl-glycero-3-phosphate; DE, 11-Deoxycortisol; CH, cholylasparagine; IA, icosanoic acid; ISO, isolinderenolide; OR, Ornithylamphotericin methyl ester; TIL, tilmicosin

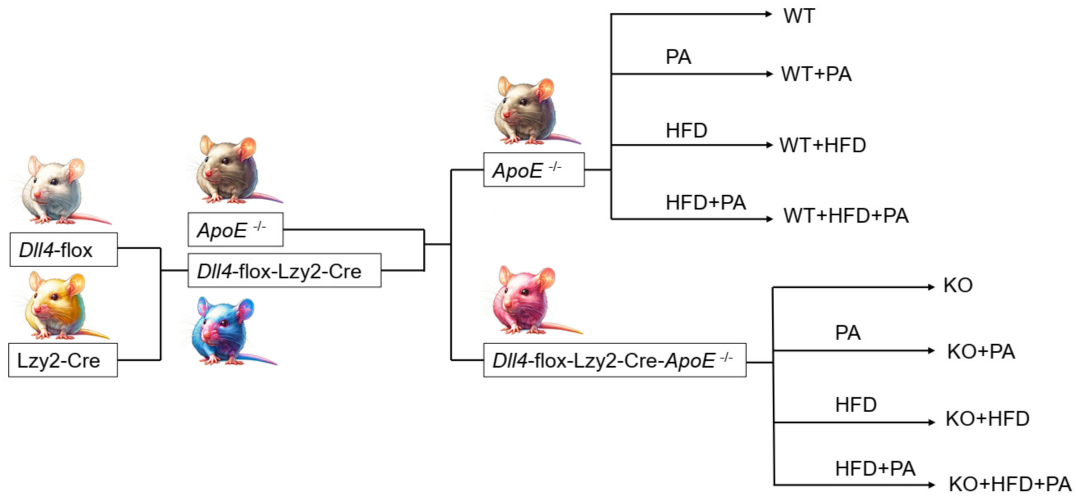

Figure S13: Flowchart illustrating the methodology for establishing both wild-type atherosclerosis (WT) and conditional Dll4 knockout atherosclerosis (KO) mouse models, along with their respective treatments.

A schematic diagram presents the steps used to produce macrophage-specific Dll4 knockout mice exhibiting ApoE deficiency [(Dll4flox/flox; Lyz2-Cre+/-; ApoE-/-) mice, abbreviated as KO]. Wild-type (WT) ApoE-deficient mice (ApoE-/- mice) were used as controls. Palmitic acid was introduced by feeding the animals a high-fat diet (HFD) and enriched with palmitic acid.

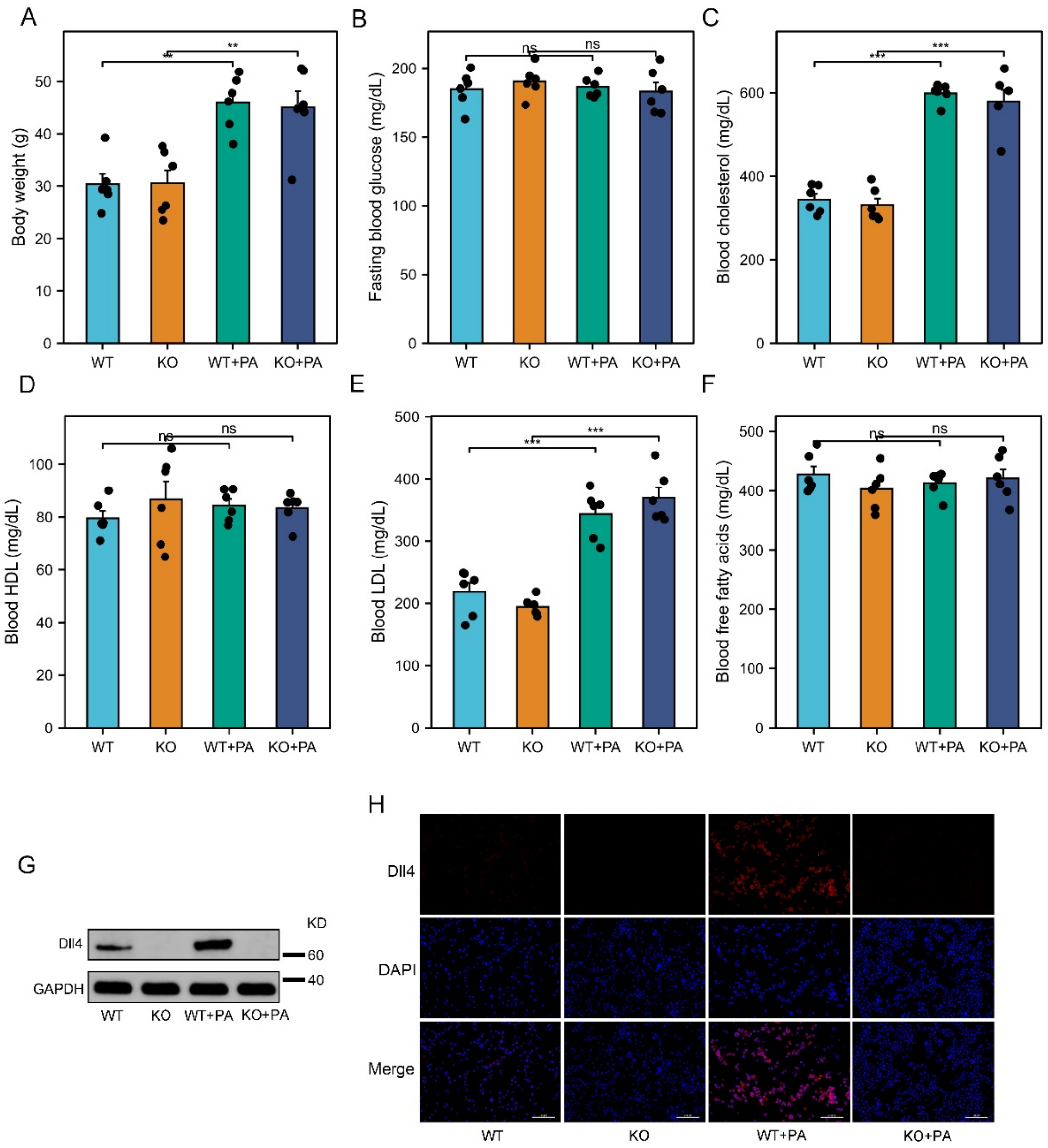

Figure S14: Impact of Palmitic Acid Administration on Dll4 Conditional Knockout Mice in an Atherosclerotic Model

This figure examines the physiological and molecular effects of palmitic acid administration in an atherosclerotic mouse model with macrophage-lineage conditional deletion of Dll4 [(Dll4$^{flox/flox}$; Lyz2-Cre$^{+/-}$; ApoE$^{-/-}$), henceforth referred to as KO mice], in comparison with their wild-type (WT) ApoE-/- counterparts. Comparative graphical representations illustrating the impact of palmitic acid administration on various physiological parameters in WT and KO mice, including bodyweight (A), fasting blood glucose concentration (B), blood cholesterol

concentration (C), blood HDL concentration (D), LDL concentration (E), and blood free fatty acid concentration (F). (ns: no significance; **P<0.01; ***P<0.001; n=6 mice). G. Representative images of Western blot assays showcasing the protein expression levels of Dll4 and the loading control GAPDH in primary macrophages isolated from WT and KO mice, both subjected to a diet enriched with palmitic acid. H. Representative immunofluorescent staining images of Dll4 in primary macrophages isolated from WT and KO mice fed with a palmitic acid-rich diet. Cell nuclei are highlighted by DAPI staining. The scale bar represents 200μm, providing a sense of cell size and staining intensity.

Abbreviations: HDL, high-density lipoprotein; LDL, low-density lipoprotein; Dll4, Delta-like ligand 4; GAPDH, Glyceraldehyde 3-phosphate dehydrogenase; DAPI, 4',6-diamidino-2-phenylindole.

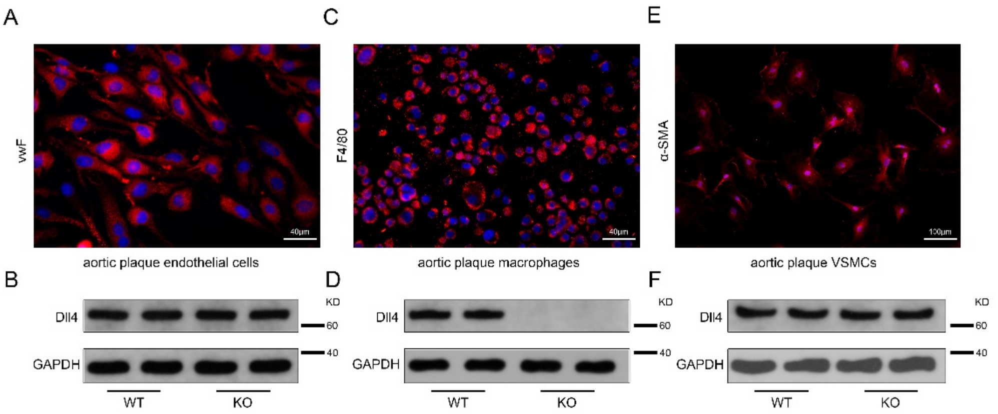

Figure S15: Assessment of Dll4 Deletion Specificity and Efficiency in Conditional KO Models

A: Confirmation of isolated primary aortic endothelial cells using vwF immunofluorescent staining. Scale bar denotes 40μm. B: Western blot analysis showcasing Dll4 expression in isolated primary aortic endothelial cells from both WT and KO models. C: Identification of isolated primary aortic macrophages via F4/80 immunofluorescent staining. Scale bar is set at 40μm. D: Immunoblot representation of Dll4 levels in primary aortic macrophages isolated from WT and KO samples. E: Validation of isolated primary aortic VSMCs throughα-SMA immunofluorescent staining. Scale bar corresponds to 100μm. F: Western blot depiction of Dll4 expression in isolated primary aortic VSMCs from both WT and KO specimens. Abbreviations: vwF, von Willebrand Factor; VSMCs, Vascular Smooth Muscle Cells; α-SMA:, α-Smooth Muscle Actin.

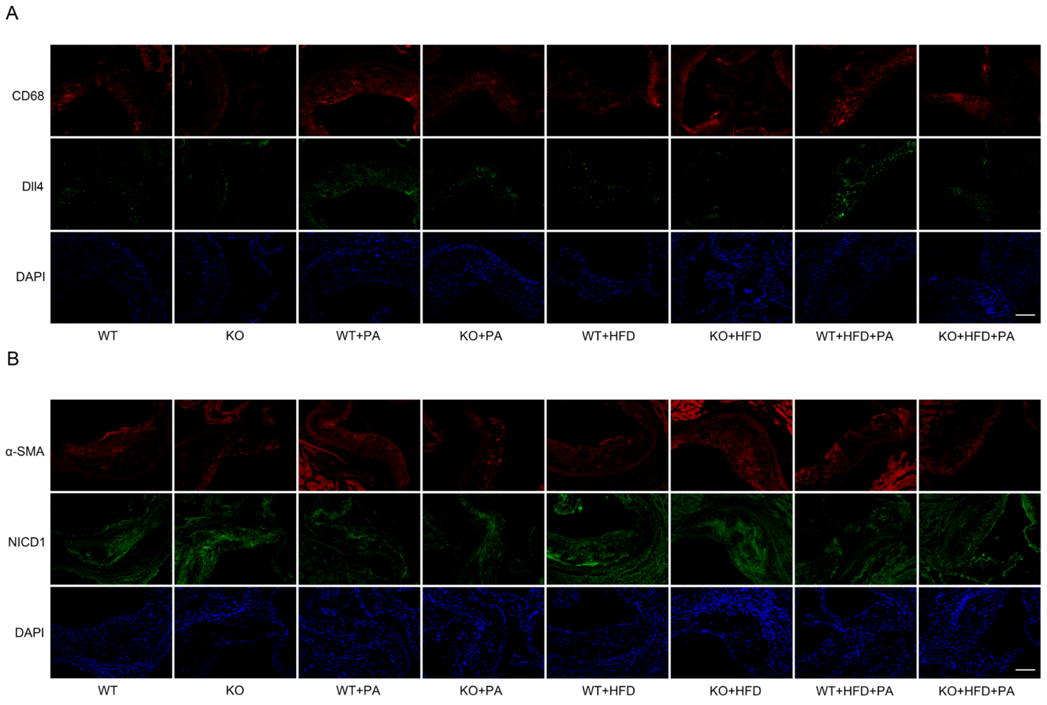

Figure S16: Separate Representative Images of Immunofluorescent Staining of Aortic Root Plaques

Presented in this figure are individual, unmerged images from the immunofluorescent staining studies, offering a comprehensive visual supplement to the data provided in Figures 5E and 5F.

A. Separate, uncombined images revealing the outcomes of dual immunofluorescent staining for CD68 and Dll4. This method uniquely illuminates the presence of M1 polarized macrophages (signaled by CD68 positivity) and concurrently traces the expression of Dll4 within these immune cells. B. Separate, uncombined images illustrating the outcome of dual immunofluorescent staining for α-SMA and NICD1. In both instances, the counterstaining of cell nuclei with DAPI enhances cellular identification and enumeration. The accompanying scale bar represents a 200μm length.

Abbreviations: α-SMA, α-Smooth Muscle Actin; NICD1, Notch Intracellular Domain 1; VSMCs, vascular smooth muscle cells; DAPI, 4',6-diamidino-2-phenylindole

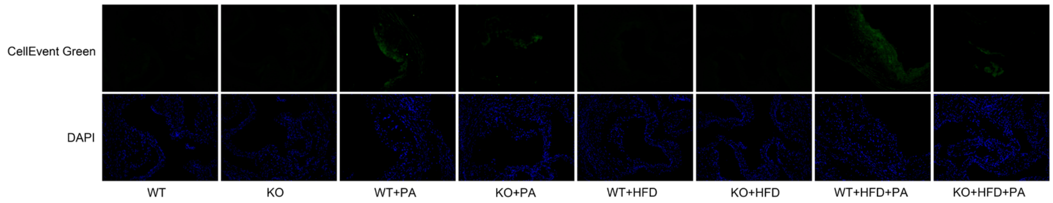

Figure S17: Individual CellEvent Green Senescence Staining Images of Aortic Root Plaques

This figure showcases distinct, standalone images derived from the CellEvent Green Senescence staining of aortic root plaques, serving as a detailed visual complement to the findings presented in Figures 5F.

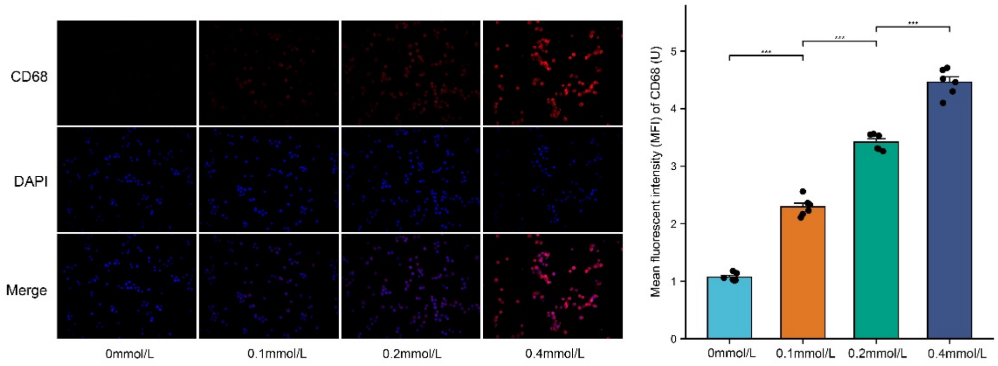

Figure S18: Effect of Palmitic Acid on M1 Polarization Induction in Primary Macrophages

The left panel displays fluorescence microscopy images showcasing CD68 staining (a marker for M1 macrophages), DAPI nuclear staining, and their merged representation for primary macrophages exposed to palmitic acid at concentrations of 0, 0.1, 0.2, and 0.4 mmol/L for a duration of 6 hours. The right panel presents bar graphs depicting the average fluorescence intensity of CD68 across the varying concentrations of palmitic acid treatments.

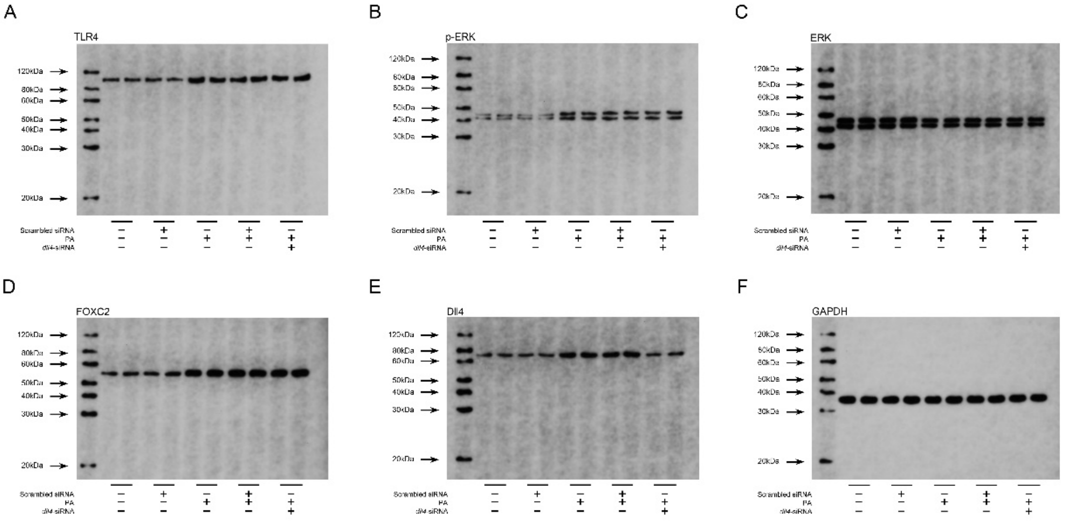

Figure S19: Uncropped Original Western Blot Gels for Targeted Proteins in Figure 6B

A. Demonstrates the TLR4 expression in macrophages subjected to palmitic acid incubation and/or Dll4 siRNA transfection. B. Exhibits the levels of phosphorylated ERK in macrophages under similar conditions. C. Depicts total ERK expression in macrophages treated with palmitic acid and/or transfected with Dll4 siRNA. D. Showcases FOXC2 expression in macrophages post palmitic acid incubation and/or Dll4 siRNA transfection. E. Reveals Dll4 expression patterns following palmitic acid treatment and/or Dll4 siRNA transfection. F. Presents GAPDH levels, used as a control, in macrophages exposed to palmitic acid and/or transfected with Dll4 siRNA. Abbreviations: TLR4, toll-like receptor 4; ERK, extracellular signal-regulated kinase; Dll4, delta-like ligand 4; FOXC2, forkhead box C2; GAPDH, glyceraldehyde 3-phosphate dehydrogenase

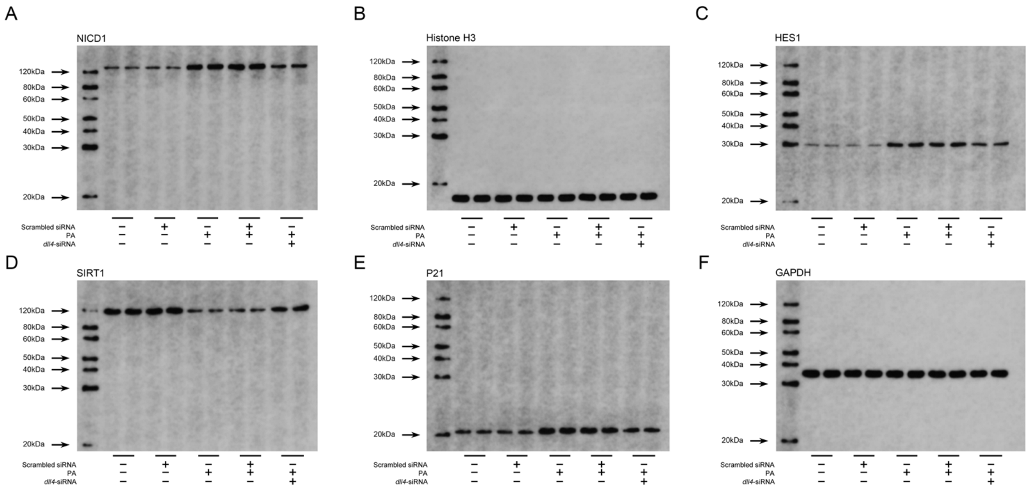

Figure S20: Presentation of Uncropped Original Western Blot Gels for Specific Proteins in Figure 6E

A. illustrates the expression of nuclear NICD1 in VSMCs co-cultured with macrophages, which have been incubated with palmitic acid and/or transfected with Dll4 siRNA, within the contact co-culture model. B. displays the levels of nuclear Histone H3 in VSMCs under analogous conditions. C. portrays the expression of HES1 in VSMCs in the same co-culture model. D. illustrates the expression of SIRT1 in VSMCs in the same co-culture model. E. unveils the expression patterns of SIRT1 in VSMCs co-cultured with similarly treated macrophages. F. presents the levels of GAPDH, utilized as a control, in VSMCs within the contact co-culture model with macrophages exposed to palmitic acid and/or transfected with Dll4 siRNA. Abbreviations: VSMCs, Vascular Smooth Muscle Cells; HES1, hairy and enhancer of split-1; SIRT1, sirtuin 1; P21, cyclin-dependent kinase inhibitor P21; NICD1, Notch Intracellular Domain 1; GAPDH, glyceraldehyde 3-phosphate dehydrogenase

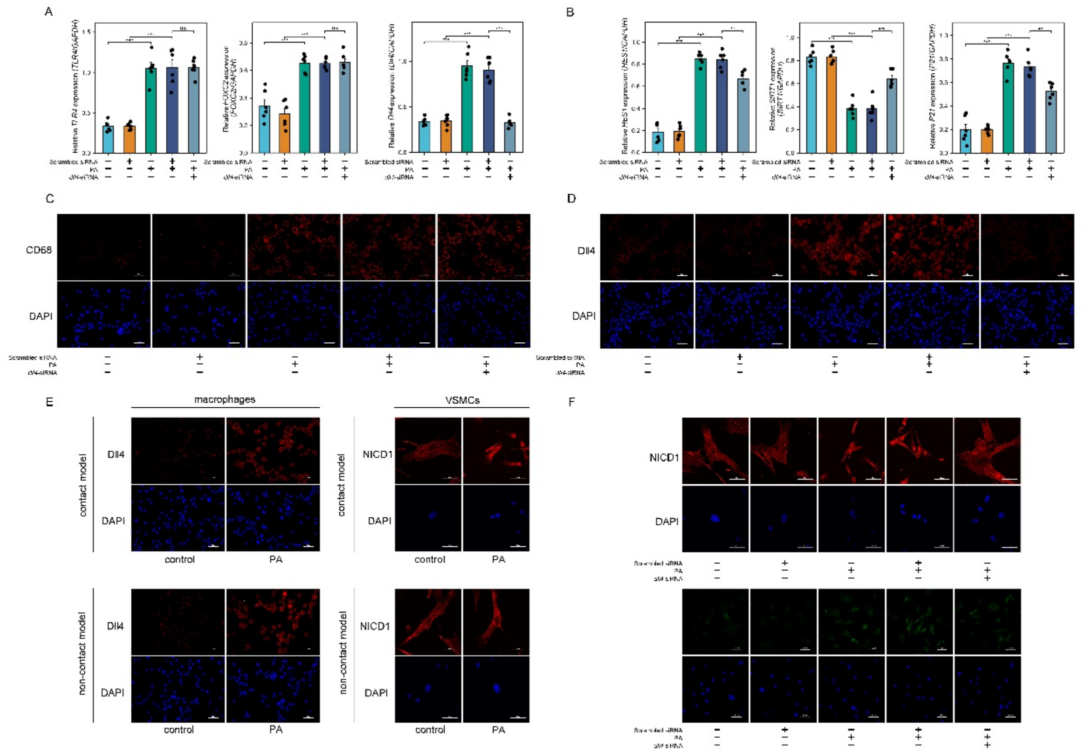

Figure S21: Influence of Macrophage Dll4 Silencing on VSMCs Notch Pathway Activation

A. Quantitative analysis of relative mRNA expression levels of *TLR4*, *FOXC2*, and *Dll4* in macrophages incubated with palmitic acid (*** P<0.001, n=6 independent replicates). B. Quantification of relative mRNA expression levels of *HES1*, *SIRT1*, and *P21* in VSMCs, when in contact co-culture with palmitic acid-treated macrophages transfected with *Dll4* small interfering RNA (siRNA) (*** P<0.001, n=6 independent replicates). C. Selected unmerged immunofluorescent staining images of CD68 in macrophages. These images serve as a supplement to Figure 6A. Cell nuclei were counterstained with DAPI. The scale bar indicates a length of 50μm. D. Representative images depicting unmerged immunofluorescent staining of Dll4 in macrophages. These images complement Figure 6A. Cell nuclei were stained with DAPI. The scale bar represents a length of 50μm. E. Selected unmerged immunofluorescent staining images showcasing Dll4 in macrophages and NICD1 in VSMCs in both contact and non-contact co-culture models. These images supplement Figure 6D. Cell nuclei were counterstained with DAPI. The scale bar denotes a length

of 50μm. F. Representative images showing unmerged immunofluorescent staining of NICD1 (scale bar=50μm) and CellEvent Green staining (scale bar=40μm) in VSMCs. These images provide additional visual data to Figure 6F. Cell nuclei were stained with DAPI.

Abbreviations: VSMCs, Vascular Smooth Muscle Cells; TLR4, Toll-like receptor 4; FOXC2, Forkhead box C2; Dll4, Delta-like ligand 4; HES1, hairy and enhancer of split-1; SIRT1, sirtuin 1; P21, cyclin-dependent kinase inhibitor P21; NICD1, Notch Intracellular Domain 1; DPAI, 4',6-diamidino-2-phenylindole

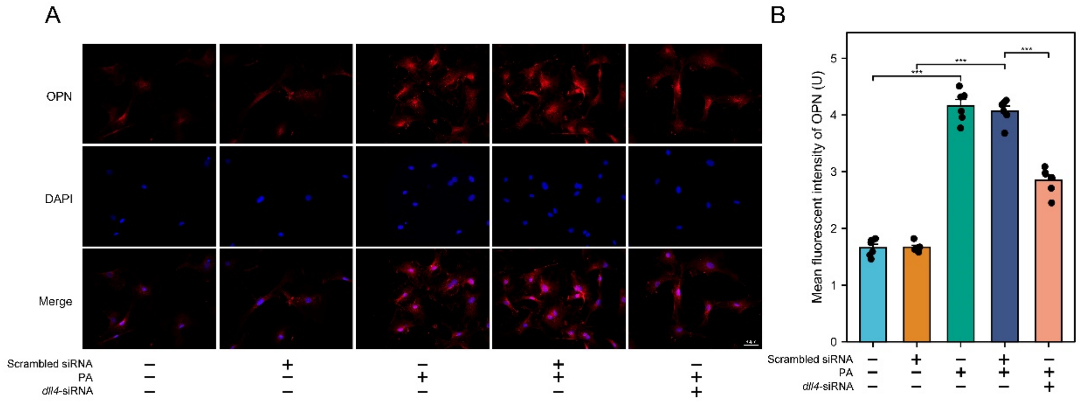

Figure S22: Effects of macrophage Dll4 silencing on palmitic acid-induced macrophage-mediated VSMCs synthetic phenotypic conversion

A: Displays representative immunofluorescent images showcasing osteopontin (OPN) expression in vascular smooth muscle cells (VSMCs) when co-cultured with macrophages subjected to palmitic acid incubation and/or Dll4 siRNA transfection in the contact co-culture setup. B: Provides a quantitative representation of the mean fluorescent intensity of OPN in VSMCs. (*** P<0.001, n=6 independent replicates)

Abbreviations: OPN, osteopontin; VSMCs, vascular smooth muscle cells; Dll4, delta-like ligand 4.

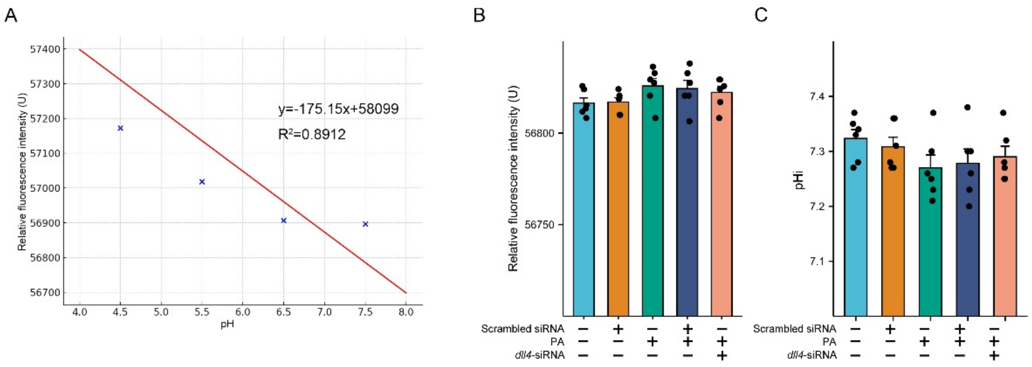

Figure S23: Intracellular pH (pHi) Assessment in VSMCs Prior to Senescence Analysis

A: Standard curve derived from the Intracellular pH Calibration Buffer Kit, facilitating the conversion of fluorescence ratios to pH values. B: Quantitative fluorescence intensity in VSMCs following staining with pHrodo Red Intracellular pH Indicator. C: Computed pHi values for VSMCs. (n=6 independent replicates)

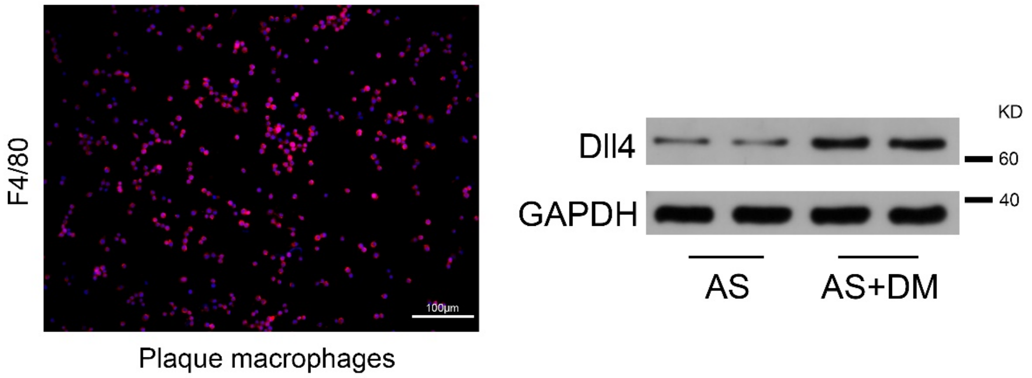

Figure S24: Assessment of Dll4 expression in macrophages isolated from aortic atherosclerotic plaques in mice

Left Panel: Confirmation of macrophage isolation from aortic atherosclerotic plaques using F4/80 immunofluorescent staining. Right Panel: Immunoblots showcasing Dll4 and GAPDH expression in macrophages derived from aortic atherosclerotic plaques of two mouse models: atherosclerosis (AS) and atherosclerosis with concomitant Type 2 Diabetes Mellitus (AS+DM).

Table S1: Criteria to determine presence of diseases in NIS database

Diseases	Diagnoses code
Coagulopathy	ICD-10-CM diagnoses code D684, D68311, D6851, D68312, D6861, D689, D6832, D682, D681, D6862, D68318, D6859, D688, D6869, D6852, D680
Obesity	ICD-10-CM diagnoses code E669, E668, E6609, E661, E6601, E662
Hypertension	ICD-10-CM diagnoses code I10, I152, I151, I158, I150, I159
Hypothyroidism	ICD-10-CM diagnoses code E034, E030, E031, E032, E039, E034, E038, E033
Coronary artery dis ease	ICD-10-CM diagnoses code I25111, I25118, I25119, I25110, I2510, I252, I255, I256, I25812, I25810, I25811, I2582, I2584, I2583, I2589, I259
Atrial fibrillation	ICD-10-CM diagnoses code I482, I4820, I4811, I4819, I480, I4821, I481, I4891
Diabetes mellitus	ICD-10-CM diagnoses code E10.x-E13.x,
Peripheral vascular disease	ICD-10-CM diagnoses code I7389, I739, I70201, I70209, I70503, I70502, I70508, I70501, I70509, I70603, I70602, I70608, I70601, I70609, I70703, I70702, I70708, I70701, I70709, I70303, I70302, I70308, I70301, I70309
Hypercholesteremia	ICD-10-CM diagnoses code E7801, E780, E7800
Alcohol use	ICD-10-CM diagnoses code F10180, F1014, F10150, F10151, F10159, F10181, F10182, F10121, F10120, F10129, F10188, F1019, F10131, F10132, F10130, F10139, F1011, F1010, F10280, F1024, F1026, F1027, F10250, F10251, F10259, F10281, F10282, F10221, F10220, F10229, F10288, F1029, F10231, F10232, F10230, F10239, F1021, F1020, F10980, F1094, F1096, F1097, F10950, F10951, F10959, F10981, F10982, F10921, F10920, F10929, F10988, F1099, F10931, F10932, F10930, F10939
Tobacco abuse	ICD-10-CM diagnoses code Z716, Z720, F17221, F17220, F17228, F17229, F17223, F17291, F17290, F17298, F17299, F17293
Cerebral infarction	ICD-10-CM diagnoses code I636, I6312, I63423, I63133, I63443, I63413, I63433, I63113, I63422, I63132, I63442, I63412, I63432, I63112, I6349, I6319, I63421, I63131, I63441, I63411, I63431, I63111, I63429, I63139, I63449, I6340, I63419, I63439, I6310, I63119, I6302, I63323, I63033, I63343, I63313, I63333, I63013, I63322, I63032, I63342, I63312, I63332, I63012, I6339, I6309, I63321, I63031, I63341, I63311, I63331, I63011, I63329, I63039, I63349, I6330, I63319, I63339, I6300, I63019, I6322, I63523, I63233, I63543, I63513, I63533, I63213, I63522,

	I63232, I63542, I63512, I63532, I63212, I6359, I6329, I63521, I63231, I63541, I63511, I63531, I63211, I63529, I63239, I63549, I6350, I63519, I63539, I6320, I63219, I639, I638, I6389, I6381
Cerebral hemorrhage	ICD-10-CM diagnoses code 'I613, I614, I611, I610, I612, I615, I616, I619, I618, I6201, I6203, I621, I629, I6202, I6200
Acute coronary syndrome	ICD-10-CM diagnoses code I201, I209, I208, I200, I220, I221, I228, I229, I2102, I2121, I2101, I2109, I2119, I2129, I2111, I213, I214, I222
Unstable angina pectoris	ICD-10-CM diagnoses code I201, I209, I208, I200
Acute ST-segment elevation myocardial infarction	ICD-10-CM diagnoses code I220, I221, I228, I229, I2102, I2121, I2101, I2109, I2119, I2129, I2111, I213
Acute non-ST elevation myocardial infarction	ICD-10-CM diagnoses code I214, I222

TableS2: Case Report Form

q1 Study Affiliation: # (1) Shaanxi Provincial People's Hospital
q2 Hospital number: _______________
q3 Name: ____________
q4 Enrollment date: <yyyy/mm/dd> (year/month/day)
q5 Visit date: <yyyy/mm/dd> (year/month/day)
q6 Date of birth: <yyyy/mm/dd> (year/month/day)
q6a Age: ### years old
q7 Gender: # (1) Male (2) Female
q8 ID number: _____________________________________
q9 Personal phone: _____________________________________
q10 Home address: ____
q11 Contact person: ___________
q12 Relationship: ___________
q13 Contact phone 1: _______________________
q14 Contact phone 2: _______________________
q15 Education level: # (1) Primary school or below (2) Junior high school (3) Technical secondary school or high school (4) University or undergraduate (5) Postgraduate
q16 Occupation: ## (1) Farmer (2) Professional (3) Civil servant (4) Police or military (5) Business person (6) Freelancer (7) Homemaker (8) Technical worker (9) Non-technical worker (10) Unemployed (11) Retired (12) Other
q17 Marital status: # (1) Single (2) Married (3) Divorced (4) Widowed
q18 Medical insurance status: # (1) New rural cooperative medical system (2) Social medical insurance (3) Public medical insurance (4) Serious illness insurance (5) Commercial medical insurance (6) Self-pay
q19 Allergy history: # (1) Yes (2) No
q19a Allergen: _______________________
q20 Coronary heart disease: # (1) Yes (2) No
q20a Course of disease: _____ years
q20b Type (multiple choices allowed): ________ (1) Asymptomatic coronary heart disease (2) Stable angina pectoris (3) Unstable angina pectoris (4) Acute ST-segment elevation myocardial infarction (5) Acute non-ST-segment elevation myocardial infarction (6) Old myocardial infarction
q20c Standardized treatment for coronary heart disease medication: # (1) Yes (2) No
q21 Hypertension: # (1) Yes (2) No
q21a Course of disease: _____ years
q21b Highest previous blood pressure: Systolic pressure ### Diastolic pressure: ### mmHg
q21c Whether hypertension is regularly treated: # (1) Yes (2) No
q22 Cardiomyopathy: # (1) Yes (2) No q22a Duration: _______ years q22b Cause: ## (1) Dilated cardiomyopathy (2) Hypertrophic cardiomyopathy (3) Restrictive cardiomyopathy (4) Arrhythmogenic right ventricular cardiomyopathy (5) Myocarditis (6) Alcoholic cardiomyopathy (7) Diabetic cardiomyopathy (8)

Peripartum cardiomyopathy
q23 Valvular heart disease-associated cardiomyopathy: # (1) Yes (2) No
q23a Duration: _______ years
q23b Cause: ## (1) Rheumatic (2) Degenerative (3) Ischemic necrosis (4) Mucoid degeneration (5) Congenital anomalies (6) Connective tissue disorders (7) Trauma (8) Uncertain
q23c Type: ## (1) Mitral stenosis (2) Mitral regurgitation (3) Aortic stenosis (4) Aortic regurgitation (5) Tricuspid stenosis (6) Tricuspid regurgitation (7) Pulmonary stenosis (8) Pulmonary regurgitation
q24 Congenital heart disease: # (1) Yes (2) No q24a Duration: _______ years q24b Type: ## (1) Atrial septal defect (2) Ventricular septal defect (3) Patent ductus arteriosus (4) Coarctation of the aorta (5) Pulmonary artery stenosis (6) Aortic valve stenosis (7) Tetralogy of Fallot (8) Ebstein anomaly
q25 Pulmonary heart disease: # (1) Yes (2) No q25a Duration: _______ years
q26 Diabetes: # (1) Yes (2) No q26a Duration: _______ years q51 Family history of diabetes: # (1) Yes (2) No
q27 Digestive system diseases: # (1) Yes (2) No q27a Duration: _______ years q27b Type: ## (1) Gastroesophageal reflux disease (2) Gastritis (3) Peptic ulcer disease (4) Biliary disease (5) Pancreatitis (6) Ulcerative colitis (7) Crohn's disease
q28 History of exposure to cardiotoxic drugs: # (1) Yes (2) No q28a Drug exposure: ___________________________
q29 Smoking history: # (1) Yes (2) No
q29a Former smoker, quit smoking for ## years
q29a1 ### cigarettes per day
q29b No addiction, occasional smoker, smoking for ## years
q29b1 ### cigarettes per day
q29c Currently smoking, smoking for ## years
q29c1 ### cigarettes per day
q30 Drinking history: # (1) Yes (2) No
q30a Quit drinking now, drank for ## years
q30a1 ### liang of baijiu per day
q30a2 ## bottles of beer per day
q30b Occasionally drink, drank for ## years
q30b1 ### liang of baijiu per day
q30b2 ## bottles of beer per day
q30c Drink frequently, drank for ## years
q30c1 ### liang of baijiu per day
q30c2 ## bottles of beer per day
q31 Family history: # (1) Yes (2) No
q31a Please mark: _____________________________
q32 Previously diagnosed type of coronary heart disease: # (1) Silent coronary heart disease (2) Stable angina (3) Unstable angina (4) Acute ST-segment elevation myocardial infarction (5) Acute non-ST-segment elevation myocardial infarction (6) Old myocardial infarction

q33 Previously diagnosed with coronary heart disease: # (1) Yes (2) No q33a History of coronary heart disease: ## years
q34 Clinical symptoms: # (1) No chest pain attacks during daily activities (2) Slightly limited in daily activities due to angina (3) Significantly limited in daily activities due to angina (4) Angina occurs during any physical labor
q35 Weight: ###.## kg
q36 Height: ###.## cm
q37 Systolic blood pressure: ### mmHg
q38 Diastolic blood pressure: ### mmHg
q39 Pulmonary moist rales: # (1) None (2) Unilateral lung base (3) Bilateral lung base (4) Unilateral diffuse (5) Bilateral diffuse
q40 Heart rate: ### beats/minute
q41 Rhythm: # (1) Regular (2) Irregular (3) Absolutely irregular
q42 External jugular vein (sitting position): # (1) Not visible (2) Filled (3) Engorged
q43 Lower limb edema: # (1) None (2) Unilateral (3) Bilateral
q44 Degree of lower limb edema: # (1) Mild (2) Moderate (3) Severe
q45 NT-proBNP: ______________pg/ml
q46 Electrocardiogram (ECG) examination: # (1) Yes (2) No
q46a ECG examination time: <yyyy/mm/dd> (year/month/day)
q46b ECG heart rate: ### beats/minute
q46c P-R interval ###.### ms
q46d QRS interval ###.### ms
q46e QT interval ###.### ms
q47 Examination result: # (1) Normal (2) Abnormal, no clinical significance (3) Abnormal, with clinical significance
q47a Please provide specific description: ______________________________
q48 Echocardiogram:
q48a Aortic ring/sinus/aortic arch: ________
q48b Right atrium (diameter): ___________
q48c Right ventricle (anterior/posterior/diameter): __________
q48d Left atrium (anterior/posterior/diameter): ___________
q48e Left ventricle (anterior/posterior/long diameter): ___________
q48f Left ventricular end-diastolic diameter: ___________
q48g Ejection fraction: ___________
q48h Ultrasound diagnosis: ____
q49 Renal function + electrolytes:
q49a Blood urea nitrogen (BUN): ##.## mmol/L
q49b Creatinine (CR): ###.## mmol/L
q49c Potassium: ##.## mmol/L
q49d Sodium: ###.### mmol/L
q49e Chloride: ###.### mmol/L
q49f Uric acid (UA): ####.## umol/L
q49g Ionized calcium: ###.### mmol/L
q49h Glucose (GLU): ##.## mmol/L

q49i Cystatin C: ##.## mmol/l
q50 Urine routine: ##.## mmol/L
q50a Urine glucose: # (1) negative (2) + (3) ++ (4) +++
q50b Urine protein: # (1) negative (2) + (3) ++ (4) +++
q50c Urine urobilinogen: # (1) negative (2) positive
q50d Urine specific gravity: ##.###
q50e Ketone: # (1) negative (2) + (3) ++ (4) +++
q50f Urine occult blood: (1) negative (2) + (3) ++ (4) +++
q50g Nitrite: # (1) negative (2) positive
q50h White blood cells: # (1) negative (2) + (3) ++ (4) +++
q51 Complete blood count:
q51a White blood cells: ##.## 109/L
q51b Neutrophils percentage: ##.## %
q51c Lymphocytes percentage: ##.##%
q51d Monocytes percentage: ##.##%
q51e Eosinophils percentage: ###.###%
q51f Basophils percentage: ###.###%
q51g Neutrophils: ###.### 109/L
q51h Lymphocytes: ##.## 109/L
q51i Monocytes: ###.### 109/L
q51j Eosinophils: ###.### 109/L
q51k Basophils: ###.### 109/L
q51l Red blood cells: ##.## 1012/L
q51m Hemoglobin: ###.## g/L
q51n Hematocrit: ###.###/L
q51o Mean corpuscular volume: ###.###fL
q51p Mean corpuscular hemoglobin: ###.###pg
q51q Mean corpuscular hemoglobin concentration: ###.###g/l
q51r Red cell distribution width: ###.###%
q51s Platelets: ###.## 109/L
q51t Plateletcrit: ###.###%
q51u Mean Platelet Volume (MPV): ##.## fl
q51v Platelet Distribution Width (PDW): ###.###%
q52 Liver Function and Lipids:
q52a Alanine Transaminase (ALT): ###.## U/L
q52b Aspartate Transaminase (AST): ###.## U/L
q52c AST/ALT ratio: ###.##
q52d Total Bilirubin: ###.###
q52e Direct Bilirubin (DBIL): ###.### ummol/L
q52f Albumin: ##.## g/l
q52g Total Cholesterol (TC): ###.## mmol/L
q52h Triglycerides (TG): ###.## mmol/L
q52i High-Density Lipoprotein (HDL-C): ##.## mmol/L
q52j Low-Density Lipoprotein (LDL-C): ##.## mmol/L

q53 Cardiac Biomarkers:
q53a Creatine Kinase (CK): ##.## ng/mL
q53b CK-MB: ##.## ng/mL
q53c Myoglobin: ##.## ng/mL
q53d High-Sensitivity Troponin T: ##.## ng/mL
q54 Coagulation Four Items + Fibrinolysis:
q55a Prothrombin Time (PT): ##.## sec
q55b PT International Normalized Ratio (INR): ##.## sec
q55c Fg：##.## g/L
q55d Plasma D-dimer: ##.## mg/L
q56 Glycated hemoglobin: ##.##%
q57 Thyroid function test:
q57a TSH: ##.## uIU/mL
q57b FT3: ##.## pmol/L
q57c FT4: ##.## pmol/L
q58 NYHA functional classification: #
(1) Class I, no symptoms with ordinary activity
(2) Class II, mild symptoms with ordinary activity (such as dyspnea or fatigue)
(3) Class III, marked limitation of activity due to symptoms, comfortable only at rest
(4) Class IV, severe limitation of activity, symptoms at rest
(5) Not classified
q59 Killip functional classification: #
(1) Class I, no clinical signs of heart failure
(2) Class II, rales or crackles in the lungs, up to half of the lung fields
(3) Class III, pulmonary edema, more than half of the lung fields
(4) Class IV, cardiogenic shock
(5) Not classified
q60 Type of coronary heart disease: #
(1) Asymptomatic coronary artery disease
(2) Stable angina pectoris
(3) Unstable angina pectoris
(4) Acute ST-segment elevation myocardial infarction
(5) Acute non-ST-segment elevation myocardial infarction
(6) Old myocardial infarction
q61 Coexisting diseases: # (1) No (2)
____
q62 Aspirin: (1) No (2) Yes
q63 Aspirin dose: ________ mg/day
q64 ADP receptor antagonists: (1) No (2) Clopidogrel (3) Ticagrelor (4) Prasugrel (5) Other (specify c13a)
q64a Other ADP receptor antagonist: ____________________
q65 ADP receptor antagonist dose: ________ mg/day
q66 Statins: (1) No (2) Atorvastatin (3) Rosuvastatin (4) Fluvastatin (5) Lovastatin (6) Simvastatin (7) Pravastatin (8) Other (specify c21a)

q67 Other statin: ____________________
q68 Statin dose: ________ mg/day
q69 Diuretics (thiazide or loop diuretics): # (1) No (2) Yes
q70 Furosemide dose: ###.## mg, ## times/day
q70a Hydrochlorothiazide dose: ###.## mg, ## times/day
q71 Date of diuretic initiation: <yyyy/mm/dd> (year/month/day)
q72 ACE inhibitors: # (1) No (2) Captopril (3) Benazepril (4) Enalapril (5) Perindopril (6) Fosinopril (7) Lisinopril (8) Ramipril (9) Trandolapril (10) Other
d72a Other ACE inhibitor: ____________________
q73 ACE inhibitor dose: ###.## mg, ## times/day
q74 Date of ACE inhibitor initiation: <yyyy/mm/dd> (year/month/day)
q75 Angiotensin II receptor blockers (ARBs): # (1) None (2) Losartan (3) Valsartan (4) Telmisartan (5) Candesartan (6) Irbesartan (7) Other (specify in d22a)
d75a Other name: ___________
q76 ARB dose: ###.##mg, ## times/day
q77 ARB start date: <yyyy/mm/dd> (year/month/day)
q78 Beta-blockers: # (1) None (2) Metoprolol (3) Bisoprolol (4) Carvedilol (5) Other (specify in d24a)
d78a Other name: ___________
q79 Beta-blocker dose: ###.##mg, ## times/day
q80 Beta-blocker start date: <yyyy/mm/dd> (year/month/day)
q81 Spironolactone: # (1) No (2) Yes
q82 Spironolactone dose: ###.##mg, ## times/day
q83 Spironolactone start date: <yyyy/mm/dd> (year/month/day)
q84 Nitrates: # (1) No (2) Yes
q85 Nitrate dose: ###.##mg, ## times/day
q86 Nitrate start date: <yyyy/mm/dd> (year/month/day)
q87 Coenzyme Q10: # (1) No (2) Yes
q88 Coenzyme Q10 dose: ###.##mg, ## times/day
q89 Coenzyme Q10 start date: <yyyy/mm/dd> (year/month/day)
q90 Hypoglycemic agents: # (1) No (2) Yes
q90a Hypoglycemic agent name: ________________________
q90b Hypoglycemic agent dose: ###.##mg, ## times/day
q90c Hypoglycemic agent start date: <yyyy/mm/dd> (year/month/day)
q91 Other drug 1: ______________________________________
q92 Other drug 1 start date: <yyyy/mm/dd> (year/month/day)
q93 Other drug 2: _______________________________________
q94 Other drug 2 start date: <yyyy/mm/dd> (year/month/day)
q95 Other drug 3: _______________________________________
q96 Other drug 3 start date: <yyyy/mm/dd> (year/month/day)
q97 Other drug 4: _______________________________________
q98 Other drug 4 start date: <yyyy/mm/dd> (year/month/day)
q99 Other drug 5: _______________________________________
q100 Other drug 5 start date: <yyyy/mm/dd> (year/month/day)

q101 Other drug 6: ____
q102 Other drug 6 start date: <yyyy/mm/dd> (year/month/day)
q103 Other drug 7: ____
q104 Other drug 7 start date: <yyyy/mm/dd> (year/month/day)

Table S3: Primer sequences for targeted genes

gene name	sequences	Size (bp)
TLR4	sense: 5'- GCCCTACCAAGTCTCAGCTA-3'	165
	antisense: 3'- CTGCAGCTCTTCTAGACCCA-5'	
FOXC2	sense: 5'- ATGTTCGAGAATGGCAGCTT-3'	171
	antisense: 3'- GACTTTCTTCTCGGCCTCCT-5'	
Dll4	sense: 5'- GTCTGCAACTGTCCTTATGGCTTTG-3'	270
	antisense: 3'- CAGCTCCTTCTTCTGGTTTGTGT-5'	
HES1	sense: 5'- CCGGCATTCCAAGCTAGAGA-3'	245
	antisense: 3'- CGTTGATCTGGGTCATGCAG-5'	
SIRT1	sense: 5'- ATCGTTACATATTCCACGGTGCT-3'	138
	antisense: 3'- CACTTTCATCTTCCAAGGGTTCT-5'	
P21	sense: 5'- AATCCTGGTGATGTCCGACCTGTT-3'	152
	antisense: 3'- ACGAAGTCAAAGTTCCACCGTTCT-5'	
GAPDH	sense: 5'-CAAGGTCATCCATGACAACTTTG-3'	496
	antisense: 5'-GTCCACCACCCTGTTGCTGTAG-3'	

Table S4: Mean signal intensities, fold change (FC), Log2 FC, VIP and P values for metabolites annotated by Lipid Maps within "Fatty Acids and Conjugates" in Chronic Stable Coronary Atherosclerotic Disease Prospective Cohort

Metabolite name	Mean signal intensities (U)		FC	Log2FC	VIP	*P* value*
	non-T2DM	T2DM				
A,b-Dihydroxyisobutyric acid	316.87	211.62	1.5	-0.582	1.52	0.00136
5-Acetamidopentanoate	113.52	76.62	1.48	-0.567	1.62	0.00312
17-Octadecynoic acid	1691.95	1332.29	1.27	-0.345	1.94	0.00025
8-Oxohexadecanoic acid	315.97	214.36	1.47	-0.56	1.42	0.02282
Cetoleic acid	55.1	35.71	1.54	-0.626	1.82	0.00046
10-Hydroxy-2,8-decadiene-4,6-diynoic acid	207.35	168.9	1.23	-0.296	1.82	0.00068
2-amino-8-oxo-9,10-epoxy-decanoic acid	1163.39	159.07	7.31	-2.871	2.61	<0.0001
5-Aminopentanoate	574.64	36.78	15.62	-3.966	2.55	<0.0001
8-Amino-7-oxononanoate	131.24	87.67	1.5	-0.582	2.23	0.00005
xi-5-Hydroxydodecanoic acid	117.39	81.55	1.44	-0.526	1.99	0.00037
3,5,7,9,11-dodecapentaenoic acid	85.13	62.27	1.37	-0.451	1.16	0.04781
2-Hydroxy-22-methyltetracosanoic acid	8783.98	9607.83	0.91	0.129	0.45	0.34412
2-Chlorooctadecanoic acid	6587.58	6459.47	1.02	-0.028	0.03	0.79167
3-methyl pyruvic acid	506.24	391.37	1.29	-0.371	1.03	0.05028
(2S,3R)-3-Hydroxy-2-methylbutanoic acid	2470.72	2922.59	0.85	0.242	0.75	0.10654
Methyl methacrylate	166.22	189.52	0.88	0.189	0.64	0.1941
3-Hexenoic acid	99.98	47.8	2.09	-1.065	1.14	0.08098

Butanoic acid	54.59	0.71	77.04	-6.268	0.99	0.14861
Valeric acid	108.54	109.48	0.99	0.012	0.06	0.96458
3-Methyl-5-pentyl-2-furanpentanoic acid	273.78	41.59	6.58	-2.719	0.91	0.11866
4-Hydroxy-2-oxohexanoic acid	245.53	193.85	1.27	-0.341	0.99	0.10595
D-Glucarate	421.9	736.32	0.57	0.803	0.33	0.47083
3-Methyl-5-pentyl-2-furanheptanoic acid	193.75	224.1	0.86	0.21	0.41	0.45091
3-Hydroxydodecanedioic acid	135.92	93.12	1.46	-0.546	0.71	0.1706
5-Aminolevulinic acid	72.53	66.75	1.09	-0.12	0.47	0.61313
Undecanedioic acid	110.9	103.92	1.07	-0.094	0.27	0.63215
Traumatic acid	918.95	885.54	1.04	-0.053	0.2	0.62151
3-Hydroxyisovalerate	1312	1683.95	0.78	0.36	0.67	0.20178
CMPF	7758.62	7426.33	1.04	-0.063	0.07	0.87143
10-hydroxy-2E-decenoic acid	3059.45	2664.47	1.15	-0.199	0.64	0.17935
3-Oxododecanoic acid	88.01	66.41	1.33	-0.406	0.84	0.15656
Stearidonic acid	194.55	196.02	0.99	0.011	0.26	0.90947
3-carboxy-4-methyl-5-pentyl-2-furanpropanoic acid	21542.62	18455.51	1.17	-0.223	1.2	0.05068
Tetradecanedioic acid	1059.76	1126.16	0.94	0.088	0.34	0.66087
Tetradecanoic acid	697.65	826.16	0.84	0.244	0.47	0.17284
3-Hydroxydodecanoic acid	466.12	452.72	1.03	-0.042	0.05	0.76115
(13Z,16Z)-Docosadienoic acid	137.53	144.87	0.95	0.075	0.17	0.69132
3,4-Dimethyl-5-pentyl-2-furantridecanoic acid	1467.75	1338.37	1.1	-0.133	0.28	0.55247
7Z,10Z-Hexadecadienoic acid	190.7	164.22	1.16	-0.216	0.46	0.30886
3-Oxohexadecanoic acid	42.79	43.03	0.99	0.008	0.08	0.94673
(9Z)-Hexadecenoic acid	151.99	141.09	1.08	-0.107	0.24	0.4949

10Z-Nonadecenoic acid	927.28	953.72	0.97	0.041	0.01	0.70821
Nonadecanoic acid	684.34	815.16	0.84	0.252	0.55	0.09998
3-Methyl-5-pentyl-2-furanpentadecanoic acid	15386.06	17188.95	0.9	0.16	0.72	0.07412
Adrenic acid	66	58.44	1.13	-0.176	0.79	0.11195
Cerebronic acid	866.52	995.03	0.87	0.2	0.33	0.28339
Itaconate	3137.3	3187.6	0.98	0.023	0.26	0.51301
2-Methylbutyrate	79.33	82.01	0.97	0.048	1.03	0.07625
(R)-3-Hydroxy-hexadecanoic acid	158.57	158.46	1	-0.001	0.19	0.99411
Cyclohexaneundecanoic acid	712.61	686.71	1.04	-0.053	0.17	0.69206
3-Methylbutanoic acid	267.33	272.76	0.98	0.029	0.67	0.23959
2-Isopropylmalic acid	51.21	0.18	285.3	-8.156	1.08	0.08878
3-Hydroxysebacic acid	1602.13	1761.49	0.91	0.137	0.53	0.33607
trans-Hex-2-enoic acid	7.77	0.16	47.94	-5.583	1.01	0.13498
3-Hydroxysuberic acid	424.47	211.77	2	-1.003	0.93	0.1702
gamma-Amino-gamma-cyanobutanoate	11.45	188.8	0.06	4.044	0.6	0.26269
Arachidonate	44.63	46.14	0.97	0.048	0	0.91187
12-amino-octadecanoic acid	2188.82	2171.21	1.01	-0.012	0.14	0.75164
3-Hydroxyoctanoate	55.02	56.68	0.97	0.043	0.08	0.80314
methyl 4-[2-(2-formyl-vinyl)-3-hydroxy-5-oxo-cyclopentyl]-butanoate	70.42	2.92	24.14	-4.593	0.99	0.12635
9Z-Eicosenoic acid	310.25	297.88	1.04	-0.059	0.61	0.20203
4,8,12,15,19,21-tetracosahexaenoic acid	1289.67	1610.81	0.8	0.321	0.56	0.23247
10,12-Tetradecadiene-4,6-diynoic acid, (E,E)-	98.14	97.7	1	-0.006	0.16	0.84063
Docosahexaenoic acid	93.53	102.43	0.91	0.131	0.56	0.4405
alpha-Linolenic acid	122.68	135.66	0.9	0.145	0.11	0.77654
3,4-Dimethyl-5-pentyl-2-furanoctanoic acid	162.95	174.51	0.93	0.099	0.04	0.71555

Trans-Hexa-dec-2-enoic acid	1113	1172.53	0.95	0.075	0.47	0.59866
(9Z,12Z,15Z)-Octadecatrienoic acid	293.43	379.03	0.77	0.369	0.78	0.06325
Tetracosatetraenoic acid (24:4n-6)	167.18	179.15	0.93	0.1	0.5	0.21321
4-[3]-ladderane-butanoic acid	868.29	879.93	0.99	0.019	0.18	0.76541
Docosatrienoic acid	123.25	129.87	0.95	0.075	0.07	0.50739
3-Methyl-5-pentyl-2-furanundecanoic acid	1833.52	1865.75	0.98	0.025	0.29	0.45467
Isovaleric acid	219.25	226.46	0.97	0.047	0.88	0.10974
Heneicosanoic acid	192.9	221.4	0.87	0.199	0.72	0.07147
16-Hydroxypalmitate	472.27	528.87	0.89	0.163	0.81	0.04544
Butyric acid	245.45	244.97	1	-0.003	0.12	0.93615
2-Aminoisobutyric acid	127.99	133.61	0.96	0.062	0.64	0.31935
palmitic acid	45.88	50.81	0.9	0.147	1.21	0.02359
Ximenic acid	2051.19	2439.65	0.84	0.25	1	0.0309
Tetracosanedioic acid	500.1	709.84	0.7	0.505	1.63	0.00323
9,10,13-Trihydroxystearic acid	233.15	283.2	0.82	0.281	1.03	0.04487
Icosanoic acid	38.23	57.55	0.66	0.59	1.27	0.00514
7,8-Diaminononanoate	43.73	56.02	0.78	0.357	1.85	0.00062
4-Hexynoic acid	43.12	56.61	0.76	0.393	1.61	0.01089
Erucic acid	105.73	125.57	0.84	0.248	1.62	0.00056
2-Aminoisobutyric acid	127.99	133.61	0.96	0.062	0.64	0.31935

[*]*P* values were calculated using the Student's t-test (two-tailed) assessed statistical significance for normally distributed data or the Mann-Whitney U test for non-normally distributed data, applying an FDR correction for multiple testing.

Table S5: Alterations of lysophosphatidylcholine and typical sphingolipids in Chronic Stable Coronary Atherosclerotic Disease Prospective Cohort identified by metabolomics profiling

Metabolite name	Regulated (T2DM vs. non-T2DM)	Fold change (FC)	Log2 (FC)	VIP	*P* value*
Lysophosphatidylcholine	Unchanged	0.80	-0.33	0.55	0.16
Galabiosylceramide (d18:1/22:0)	Down	2.22	1.15	0.01	1.44
Isoglobotrihexosylceramide	Unchanged	0.92	-0.12	0.10	0.54
Galabiosylceramide (d18:1/26:1(17Z))	Unchanged	1.09	0.13	0.68	0.23
Trihexosylceramide (d18:1/16:0)	Down	1.40	0.48	1.39	0.002
Galabiosylceramide (d18:1/18:0)	Unchanged	0.89	-0.16	0.49	0.21
Sphingosine-1-phosphate	Unchanged	0.94	-0.08	0.49	0.27
Phytosphingosine	Unchanged	0.97	-0.04	0.74	0.15
C16 Sphingosine	Up	0.76	-0.40	3.07	<0.001
Hexadecasphingosine	Unchanged	0.87	-0.19	0.86	0.11
Glucosylsphingosine	Unchanged	1.18	0.25	0.24	0.45

**P* values were calculated using the Student's t-test (two-tailed) assessed statistical significance for normally distributed data or the Mann-Whitney U test for non-normally distributed data, applying an FDR correction for multiple testing.
