# Supplementary material for: Palmitic acid in type 2 diabetes mellitus promotes atherosclerotic plaque vulnerability via macrophage Dll4 signaling
Source: Nat Commun. 2024 Feb 12;15:1281. doi: 10.1038/s41467-024-45582-8 (PMC10861578; doi:10.1038/s41467-024-45582-8)
Supplement: Supplementary file 3 — Reporting Summary [file 41467_2024_45582_MOESM3_ESM.pdf]

nature portfolio

Corresponding author(s): Zhongwei Liu, Yong Zhang, Shuo Pan, Junkui Wang

Last updated by author(s): Jan 15, 2024

Reporting Summary

Nature Portfolio wishes to improve the reproducibility of the work that we publish. This form provides structure for consistency and transparency in reporting. For further information on Nature Portfolio policies, see our Editorial Policies and the Editorial Policy Checklist.

Statistics

For all statistical analyses, confirm that the following items are present in the figure legend, table legend, main text, or Methods section.

n/a	Confirmed	
☐	☒	The exact sample size (n) for each experimental group/condition, given as a discrete number and unit of measurement
☐	☒	A statement on whether measurements were taken from distinct samples or whether the same sample was measured repeatedly
☐	☒	The statistical test(s) used AND whether they are one- or two-sided<br>*Only common tests should be described solely by name; describe more complex techniques in the Methods section.*
☐	☒	A description of all covariates tested
☐	☒	A description of any assumptions or corrections, such as tests of normality and adjustment for multiple comparisons
☐	☒	A full description of the statistical parameters including central tendency (e.g. means) or other basic estimates (e.g. regression coefficient) AND variation (e.g. standard deviation) or associated estimates of uncertainty (e.g. confidence intervals)
☐	☒	For null hypothesis testing, the test statistic (e.g. F, t, r) with confidence intervals, effect sizes, degrees of freedom and P value noted<br>*Give P values as exact values whenever suitable.*
☒	☐	For Bayesian analysis, information on the choice of priors and Markov chain Monte Carlo settings
☒	☐	For hierarchical and complex designs, identification of the appropriate level for tests and full reporting of outcomes
☐	☒	Estimates of effect sizes (e.g. Cohen's d, Pearson's r), indicating how they were calculated

Our web collection on statistics for biologists contains articles on many of the points above.

Software and code

Policy information about availability of computer code

Data collection	CAAS software (5.10); iReview software (2.0); iLab™ POLARIS Multi-Modality Guidance System (2.8.1); MassLynx software (4.2); Progenesis QI software (2.0)
Data analysis	Progenesis QI software (2.0); ImageJ software (1.53t); SPSS Statistics software (19.0); XCMS package in R software (4.0.3)

For manuscripts utilizing custom algorithms or software that are central to the research but not yet described in published literature, software must be made available to editors and reviewers. We strongly encourage code deposition in a community repository (e.g. GitHub). See the Nature Portfolio guidelines for submitting code & software for further information.

Data

Policy information about availability of data

All manuscripts must include a data availability statement. This statement should provide the following information, where applicable:

- Accession codes, unique identifiers, or web links for publicly available datasets
- A description of any restrictions on data availability
- For clinical datasets or third party data, please ensure that the statement adheres to our policy

The publicly available Lipid Maps database (https://lipidmaps.org/), HMDB database (https://hmdb.ca/) and KEGG compound database (https://www.genome.jp/kegg/compound/) were used in this study. The data supporting the findings of this study are available within the article and its Supplementary Information files and source data file. Source data are provided with this paper(https://doi.org/10.6084/m9.figshare.24328507.)

Research involving human participants, their data, or biological material

Policy information about studies with human participants or human data. See also policy information about sex, gender (identity/presentation), and sexual orientation and race, ethnicity and racism.

Reporting on sex and gender	The findings of the study apply to both sexes. Sex and gender were considered in the study design, ensuring a diverse representation of participants. Sex and gender determination was based on self-reporting by the participants during the initial data collection phase. The individual-level data is shared only when consent has been obtained. For our first cohort study based NIS database, numbers and percentage of female were 8224010 (60.%) and 2272628 (49.5%) in non-T2DM and T2DM groups respectively. In our second cohort study, numbers and percentage of female were 12 (21.8%) and 9 (19.6%).The details of this analysis are presented in Tables 1 and 2 of our manuscript. Our data indicate that there were no statistically significant differences in gender composition between the study groups. Findings in this study are not related to one sex.
Reporting on race, ethnicity, or other socially relevant groupings	In our first cohort study based on NIS database, we reported race in Table1. The information of race was sourced from NIS database. No ethnicity or other socially relevant groupings were involved in this study.
Population characteristics	Please see Table1 "Demographics of patients with and without T2DM in National Inpatient Sample (NIS) 2016-2018" and Table 2 "Baseline characteristics of the patients with stable coronary artery heart disease".
Recruitment	In our study examining diabetes-associated atherosclerosis, participants were recruited from the Cardiology department of the Shaanxi Provincial People's Hospital. We included individuals aged 18 to 75 with a confirmed diagnosis of Type 2 Diabetes Mellitus. Exclusion criteria encompassed patients with other significant comorbid conditions, like severe kidney disease or active cancer. Potential participants were identified through clinic records and approached during their routine visits. After explaining the study's purpose, procedures, and potential risks, written informed consent was obtained from each participant. An initial medical examination and blood tests were conducted to confirm eligibility. Given the nature and methodology of our observational study, where follow-up with participants was conducted primarily through telephone calls, regular outpatient follow-up visits, or electronic hospital records, we did not provide compensation to participants. This decision was based on the minimal burden and inconvenience placed on participants, as the study did not require significant time commitment or travel beyond their usual medical care routine. This approach aligns with our ethical considerations and guidelines for observational studies. The study protocol was approved by the hospital's Institutional Review Board, ensuring adherence to ethical guidelines and participant confidentiality.
Ethics oversight	All procedures and protocols involving human participants in this study were conducted following the principles of the Declaration of Helsinki and were approved by Shaanxi Provincial People's Hospital Institutional Review Board. Informed written consent was obtained from all individuals before their inclusion in the study. The privacy and confidentiality of all participants were rigorously maintained.

Note that full information on the approval of the study protocol must also be provided in the manuscript.

Field-specific reporting

Please select the one below that is the best fit for your research. If you are not sure, read the appropriate sections before making your selection.

☒ Life sciences ☐ Behavioural & social sciences ☐ Ecological, evolutionary & environmental sciences

For a reference copy of the document with all sections, see nature.com/documents/nr-reporting-summary-flat.pdf

Life sciences study design

All studies must disclose on these points even when the disclosure is negative.

Sample size	According to the CLEAR Outcomes trial, the MACE incidence for patients with cardiovascular diseases complicated with or without diabetes were 14.2% and 11.9% respectively [1]. Using a standard formula for comparing two proportions and setting the alpha level at 0.05 for a two-sided test, our calculations, adjusted for an estimated dropout rate, indicated that a sample size of approximately 50 per group would be sufficient to detect the expected difference in MACE incidence with a power of around 70%. Moreover, this sample size also aligns with our previous experience [2]. This decision provided a balance between statistical power and practical considerations such as participant availability and resource constraints. Exact sample size are recorded in the paper. For animal study, based on the literature and our previous studies, for each experiment we used at least n = 3 replicates to calculate the statistical values for each analysis.<br>References:<br>[1] Kausik KR, Stephen JN, Na Li, et al. Efficacy and safety of bempedoic acid among patients with and without diabetes: prespecified analysis of the CLEAR Outcomes randomised trial [J]. Lancet Diabetes Endocrinol, 2024, 12(1): 19-28<br>[2] Zhongwei Liu, Haitao Zhu, Yanpeng Ma, et al. AGEs exacerbates coronary microvascular dysfunction in NoCAD by activating endoplasmic reticulum stress-mediated PERK signaling pathway [J]. Metabolism, 2021, 117: 154710
Data exclusions	No data were excluded.
Replication	We confirm all attempts at replication were successful. The experimental findings were repeated and our data are based on at least three independent experiments with similar results unless otherwise noted in the manuscript. Details are described in the legends of the corresponding figures.

Randomization	All samples were randomly allocated into experimental groups.
Blinding	In our population cohort study, blinding was not applicable due to its observational nature, where data were derived from existing records and self-reported information, making participant and researcher blinding impractical and irrelevant to the study's methodology. For animal study, the investigators were blinded for the animal allocation to the experimental groups. All data collection and analyses were performed in a blind manner. For in vitro study, blinding was not applicable because the investigator in charge of an experiment was responsible for cell culture, treatment, sample collection, and data analysis.

Reporting for specific materials, systems and methods

We require information from authors about some types of materials, experimental systems and methods used in many studies. Here, indicate whether each material, system or method listed is relevant to your study. If you are not sure if a list item applies to your research, read the appropriate section before selecting a response.

Materials & experimental systems

n/a	Involved in the study	
☐	☒	Antibodies
☒	☐	Eukaryotic cell lines
☒	☐	Palaeontology and archaeology
☐	☒	Animals and other organisms
☐	☒	Clinical data
☒	☐	Dual use research of concern
☒	☐	Plants

Methods

n/a	Involved in the study	
☒	☐	ChIP-seq
☐	☒	Flow cytometry
☒	☐	MRI-based neuroimaging

Antibodies

Antibodies used

Antibodies for immunofluorescent stain:
Anti-CD68 antibody, Cell Signaling Technology, 29176SF, Product Clone Name: E3O7V, 1:200 dilution
Anti-Dll4 antibody, Cell Signaling Technology, 96406S, Product Clone Name: D7N3H, 1:200 dilution
Anti- NICD1 antibody, Cell Signaling Technology, 3608S, Product Clone Name: D1E11, 1:200 dilution
Anti- OPN antibody, Cell Signaling Technology, 27927S, Product Clone Name: E4O2F, 1:200 dilution
Alexa Fluor 594 conjugated secondary antibody, Jackson ImmunoResearch, 611585215, 1:1000 dilution

Antibodies for Western Blots:
Anti- TLR4 antibody, Cell Signaling Technology, 14358S, Product Clone Name: D8L5W, 1:1000 dilution
Anti- FOXC2 antibody, Cell Signaling Technology, 12974S, Product Clone Name: D4D4, 1:1000 dilution
Anti- Dll4 antibody, Cell Signaling Technology, 96406S, Product Clone Name: D7N3H, 1:1000 dilution
Anti- phosphorylated ERK antibody, Cell Signaling Technology, 4370S, Product Clone Name: D13.14.4E, 1:1000 dilution
Anti- ERK antibody, Cell Signaling Technology, 4695S, Product Clone Name: 137F5, 1:1000 dilution
Anti- HES1 antibody, Cell Signaling Technology, 11988S, Product Clone Name: D6P2U, 1:1000 dilution
Anti- SIRT1 antibody, Cell Signaling Technology, 9475S, Product Clone Name: D1D7, 1:1000 dilution
Anti- P21 antibody, Abcam, ab109520, Product Clone Name: EPR362, 1:1000 dilution
Anti- NICD1 antibody, Cell Signaling Technology, 3608S, Product Clone Name: D1E11, 1:1000 dilution
Anti- GAPDH antibody, Sigma-Aldrich, G9545, Product Clone Name: NA.41, 1:5000 dilution
Anti- Histone H3 antibody, Cell Signaling Technology, 4499S, Product Clone Name: D1H2, 1:1000 dilution
HRP- conjugated secondary antibody, Jackson ImmunoResearch, 111035045, 1:5000 dilution

Validation

All antibodies used in this study were commercially purchased and have been validated by the vendors of species and application. Validation data are available from the respective vendor's respective websites.

Anti-CD68 antibody:
https://www.cellsignal.com/products/primary-antibodies/cd68-e3o7v-rabbit-mab-bsa-and-azide-free/29176

Anti-Dll4 antibody:
https://www.cellsignal.com/products/primary-antibodies/dll4-d7n3h-rabbit-mab/96406

Anti- NICD1 antibody:
https://www.cellsignal.com/products/primary-antibodies/notch1-d1e11-xp-rabbit-mab/3608

Anti-OPN antibody:
https://www.cellsignal.com/products/primary-antibodies/osteopontin-spp1-e4o2f-rabbit-mab/27927

	Alexa Fluor 594 conjugated secondary antibody:<br>https://www.jacksonimmuno.com/catalog/products/611-585-215<br><br>Anti-TLR4 antibody:<br>https://www.cellsignal.com/products/primary-antibodies/toll-like-receptor-4-d8l5w-rabbit-mab/14358<br><br>Anti-FOXC2 antibody:<br>https://www.cellsignal.com/products/primary-antibodies/foxc2-d4d4-rabbit-mab/12974<br><br>Anti- phosphorylated ERK antibody:<br>https://www.cellsignal.com/products/primary-antibodies/phospho-p44-42-mapk-erk1-2-thr202-tyr204-d13-14-4e-xp-rabbit-mab/4370<br><br>Anti- ERK antibody:<br>https://www.cellsignal.com/products/primary-antibodies/p44-42-mapk-erk1-2-137f5-rabbit-mab/4695<br><br>Anti- HES1 antibody:<br>https://www.cellsignal.com/products/primary-antibodies/hes1-d6p2u-rabbit-mab/11988<br><br>Anti- SIRT1 antibody:<br>https://www.cellsignal.com/products/primary-antibodies/sirt1-d1d7-rabbit-mab/9475<br><br>Anti- P21 antibody:<br>https://www.abcam.com/products/primary-antibodies/p21-antibody-epr362-ab109520.html<br><br>Anti- GAPDH antibody:<br>https://www.sigmaaldrich.com/US/en/product/sigma/g9545<br><br>Anti- Histone H3 antibody:<br>https://www.cellsignal.com/products/primary-antibodies/histone-h3-d1h2-xp-rabbit-mab/4499<br><br>HRP- conjugated secondary antibody:<br>https://www.jacksonimmuno.com/catalog/products/111-035-045

Animals and other research organisms

Policy information about studies involving animals; ARRIVE guidelines recommended for reporting animal research, and Sex and Gender in Research

Laboratory animals	The study involved laboratory animals, specifically mice. The species used were ApoE-deficient mice (B6/JGpt-Apoeem1Cd82/Gpt, Strain NO.T001458, ApoE-/- mice), mice with floxed Dll4 alleles (B6/JGpt-Dll4em1Cflox/Gpt, Strain NO. T009877, Dll4flox/flox mice), and Lyz2-Cre mice (B6/JGpt-Lyz2em1Cin(CreERT2)/Gpt, Strain NO.T052789). The mice were used for experiments starting at 8 weeks of age.
Wild animals	The study did not involve use of wild animals.
Reporting on sex	8-week-old male ApoE-/- mice and ApoE-/- mice received STZ injections were fed with HFD for 20 weeks. These animals were used for metabolomics analysis. 8-week-old male ApoE-/- mice, (Dll4flox/flox; Lyz2-Cre+/-; ApoE-/-) mice were fed with standard chow, HFD or HFD containing 5% palmitic acid for 20 weeks. These animals were used for investigating impact of palmitic acid on plaque vulnerability.
Field-collected samples	The study did not involve the use of samples collected from the field.
Ethics oversight	In terms of animal use, all experimental procedures were performed according to the guidelines outlined by Shaanxi Provincial People’s Hospital Institutional Review Board. The study protocol was reviewed and approved by the same committee. Every effort was made to minimize suffering and reduce the number of animals used. Euthanasia, when necessary, was performed in a humane manner according to the recommendations of the American Veterinary Medical Association (AVMA).

Note that full information on the approval of the study protocol must also be provided in the manuscript.

Clinical data

Policy information about clinical studies

All manuscripts should comply with the ICMJEguidelines for publication of clinical research and a completedCONSORT checklist must be included with all submissions.

Clinical trial registration	NCT05270330

Study protocol	https://ichgcp.net/clinical-trials-registry/NCT05270330
Data collection	Clinical data for this study were primarily collected at the Shaanxi Provincial People's Hospital, a leading healthcare institution located in Xi'an, Shaanxi Province, China. As a prominent tertiary care hospital, it offers a comprehensive range of medical services and serves as a major referral center for patients across the province and beyond. Patient recruitment commenced in January 2020 and concluded in December 2021. During this period, patients visiting Shaanxi Provincial People's Hospital and meeting the study's inclusion criteria were approached for participation. Informed consent was obtained from all participants before enrolling them in the study.
Outcomes	In our comprehensive study conducted at Shaanxi Provincial People's Hospital, patients were meticulously monitored at two distinct time points: at baseline and then 6 months after their discharge. The follow-up process was multifaceted, encompassing telephone calls, routine outpatient visits, and a thorough review of electronic hospital records. The primary objective or endpoint we aimed to assess was all-cause mortality. However, to gain a broader understanding of the patients' health outcomes, we also established several second ary endpoints. These included the recurrence of acute coronary syndrome (ACS), any emergent need for percutaneous coronary intervention (PCI), the sudden requirement for coronary artery bypass grafting, and the onset of acute cerebral infarction. Our commitment to rigorous patient monitoring and consistent communication ensured that our study achieved a remarkable 100% follow-up rate, with no instances of patients being lost to follow-up, thereby enhancing the reliability and comprehensiveness of our findings.

Plants

Seed stocks	n/a
Novel plant genotypes	n/a
Authentication	n/a

Flow Cytometry

Plots

Confirm that:

- [x] The axis labels state the marker and fluorochrome used (e.g. CD4-FITC).
- [x] The axis scales are clearly visible. Include numbers along axes only for bottom left plot of group (a 'group' is an analysis of identical markers).
- [x] All plots are contour plots with outliers or pseudocolor plots.
- [x] A numerical value for number of cells or percentage (with statistics) is provided.

Methodology

Sample preparation	Primary macrophages, isolated from mice via peritoneal lavage following intraperitoneal injection with 3% Brewer thioglycolate medium, served as the biological source for flow cytometry. Four days post-injection, the mice were euthanized, and their peritoneal exudates were collected by injecting 8 ml of ice-cold phosphate-buffered saline (PBS) into the peritoneal cavity. After centrifugation, the cell pellet was resuspended in DMEM supplemented with 10% FBS and antibiotics. These cells were then seeded in tissue culture plates, allowing them to adhere for 2 hours at 37°C in a 5% CO2 atmosphere. Following adherence, non-adherent cells were discarded, and the remaining macrophages were treated with varying concentrations of palmitic acid to induce M1 polarization and potential apoptosis. After a 6-hour incubation with palmitic acid, the macrophages were stained using an Annexin V-FITC/propidium iodide (PI) apoptosis detection kit as per the manufacturer's instructions. The stained cells were subsequently analyzed via flow cytometry to assess the extent of apoptosis, with results interpreted based on the fluorescence intensity of Annexin V-FITC and propidium iodide.
Instrument	BECKMAN, CytoFLEX
Software	CytExpert software was used for analysis
Cell population abundance	10000 cells were analyzed in each sample
Gating strategy	Forward and lateral scattering (FSC-A versus SSC-A) was used to identify the cell population of interest. For flow cytometry analysis of Annexin V-PI staining, apoptotic cells were gated using appropriate channels by gating unstained cells and single stained cells.

- [x] Tick this box to confirm that a figure exemplifying the gating strategy is provided in the Supplementary Information.
